# Does expert knowledge improve automatic probabilistic classification of gait joint motion patterns in children with cerebral palsy?

**DOI:** 10.1371/journal.pone.0178378

**Published:** 2017-06-01

**Authors:** Tinne De Laet, Eirini Papageorgiou, Angela Nieuwenhuys, Kaat Desloovere

**Affiliations:** 1 Faculty of Engineering Science, KU Leuven, Belgium; 2 Department of Rehabilitation Sciences, Faculty of Kinesiology and Rehabilitation Sciences (FaBeR), KU Leuven, Belgium; 3 Clinical Motion Analysis Laboratory, CERM, University Hospitals Leuven, UZ Leuven, Belgium; Boston Children's Hospital / Harvard Medical School, UNITED STATES

## Abstract

**Background:**

This study aimed to improve the automatic probabilistic classification of joint motion gait patterns in children with cerebral palsy by using the expert knowledge available via a recently developed Delphi-consensus study. To this end, this study applied both Naïve Bayes and Logistic Regression classification with varying degrees of usage of the expert knowledge (expert-defined and discretized features). A database of 356 patients and 1719 gait trials was used to validate the classification performance of eleven joint motions.

**Hypotheses:**

Two main hypotheses stated that: (1) Joint motion patterns in children with CP, obtained through a Delphi-consensus study, can be automatically classified following a probabilistic approach, with an accuracy similar to clinical expert classification, and (2) The inclusion of clinical expert knowledge in the selection of relevant gait features and the discretization of continuous features increases the performance of automatic probabilistic joint motion classification.

**Findings:**

This study provided objective evidence supporting the first hypothesis. Automatic probabilistic gait classification using the expert knowledge available from the Delphi-consensus study resulted in accuracy (91%) similar to that obtained with two expert raters (90%), and higher accuracy than that obtained with non-expert raters (78%). Regarding the second hypothesis, this study demonstrated that the use of more advanced machine learning techniques such as automatic feature selection and discretization instead of expert-defined and discretized features can result in slightly higher joint motion classification performance. However, the increase in performance is limited and does not outweigh the additional computational cost and the higher risk of loss of clinical interpretability, which threatens the clinical acceptance and applicability.

## Introduction

The most common physical disability in children is cerebral palsy (CP). The prevalence of this neuromotor disorder is estimated at 2.11 per 1000 live births [1]. The motor symptoms associated with CP, including spasticity, weakness, impaired balance, and loss of selective motor control, affect the child’s ability to walk. Because of these different motor symptoms, the variability with which CP affects gait is considerable. To evaluate this variety of different joint motion patterns, three-dimensional motion analysis (3DGA) is typically performed. 3DGA provides a highly-detailed assessment of joint angles, joint moments, and power during walking. The difficulty with using this comprehensive biomechanical measurement of gait is the clinical interpretation of the vast amount of multidimensional data that it generates. Summarizing this vast amount of data, for instance using gait classification [2,3], can facilitate clinical decision-making [4,5]. Nieuwenhuys et al. [6] highlighted additional advantages of gait classification: “Apart from research applications, gait classifications can improve communication among health care workers by providing a tool for describing, evaluating, and comparing gait between and among patients or groups of patients. Ultimately, it could aid lecturers teaching about gait in CP, serve as a tool for assessing treatment outcome, and potentially lead to a more in-depth understanding of the neurological cause of specific joint motion patterns, which may be associated with specific treatment indications.”

Gait classification in CP based on 3DGA data is subject to different challenges. Dobson et al. [2] defined two approaches for gait classification: qualitative and quantitative. According to qualitative approaches “decisions to group members rely on the judgment and experience of those making the decisions”. Quantitative approaches use machine learning techniques to pre-process and classify 3D gait data. Qualitative approaches optimally rely on expert knowledge but are limited by their subjective nature and inconsistency [2,7,8]. Quantitative approaches on the other hand are objective and powerful when it comes to analysing complex data, however, obtaining clinically relevant results and incorporating expert knowledge at the same time is often challenging [8]. In particular quantitative classification approaches have the risk of producing classes or classification rules for which the clinical interpretation is not straightforward [2].

An additional challenge for gait classification in children with CP is that the inter-subject variability is so high that often the observed joint motions do not match 100% with a set of pre-defined joint motion patterns [8]. Forcing CP gait to fit into one joint motion pattern, a so-called hard assignment, jeopardizes the clinical meaning [8]. This can be avoided by applying probabilistic classification approaches, which have the ability to do a soft-assignment as they calculate the probability of an observed joint motion belonging to all different joint motion patterns.

By incorporating expert knowledge into an automatic probabilistic classification of joint motions observed in the gait of children with CP, the advantages of qualitative and quantitative approaches could be combined. From the plethora of information provided by 3DGA, clinical experts can highlight the essential, clinically meaningful parts, thereby providing the quantitative approaches with a more clinically relevant subset of the available data. Unsupervised quantitative classification has the risk of resulting in classes without clinical meaning. This issue is alleviated by using a supervised classification approach that forces the outcome of the classification in classes that are expert-defined and, thus, by definition relevant to clinicians. Recently, a consensus-based gait classification supported by clinicians, including definitions of joint motion patterns and the 3DGA features characterizing these joint motion patterns, was proposed by Nieuwenhuys et al. [6]. The inter- and intra-rater reliability of this new classification was shown to be high [9]. Moreover, Statistical Parametric Mapping (SPM) was used to study the differences between these consensus-based gait classifications [10]. As such, we can state that a qualitative, clinically accepted, and validated classification of joint motion gait patterns in children with CP is currently available. This new study aimed at evaluating quantitative, probabilistic classification approaches that use this new expert knowledge provided by the Delphi-consensus study of Nieuwenhuys et al. [6].

Many quantitative approaches have been developed for the classification of joint motion gait patterns in children with CP [8], but few manage to incorporate patient- or pathology-specific clinical expert knowledge [2,3,8]. Two notable exceptions that use a Bayesian probabilistic approach were provided by Van Gestel et al. [8] and by Zhang et al. [11], showing proof-of-concept using automatic probabilistic algorithms. With respect to Zhang et al. [11], where Bayesian classification was used as a paradigm for probabilistic decision-making, the current study had several novel contributions. Firstly, Zhang et al. [11] only differentiated normal healthy gait from spastic diplegic patients while the current study considered eleven different joint motions across multiple joint motion patterns (see Table 1; ranging from three patterns for the sagittal and transverse hip joint motion and foot progression angle up to six patterns for the pelvis, and knee during swing in the sagittal plane). Secondly, Zhang et al. [11] only used four features (stride length, cadence, leg length, and age), while the current study used 23 expert-defined features (Table 2). Thirdly, the population studied by Zhang et al. [11] was rather limited (68 normal healthy individuals and 88 with the spastic diplegia form of CP), while we studied 356 patients, with a total of 1,719 gait trials.

**Table 1:** Joint motion pattern descriptions and their frequency in the dataset.

	**(%)**
**SAGITTAL PLANE**	
**Pelvis**	
PS0—Normal pelvic motion/posture–no or minor gait deviations	16.3
PS1—Increased range of motion	29.4
PS2—Increased anterior tilt on average	16.0
PS3—Increased anterior tilt and increased range of motion	35.8
PS4—Decreased anterior tilt (posterior tilt)	1.4
PS5—Decreased anterior tilt (posterior tilt) and increased range of motion	1.0
**Hip**
HS0—Normal hip motion–no or minor gait deviations	55.4
HS1—Hip extension deficit	27.5
HS2—Continuous excessive hip flexion	17.1
**Knee during stance**	
KSTS0—Normal knee motion during stance–no or minor gait deviations	15.9
KSTS1—Increased knee flexion at initial contact	8.1
KSTS2—Increased knee flexion at initial contact and earlier knee extension movement		25.4
KSTS3—Knee hyperextension	8.0
KSTS4—Knee hyperextension and increased knee flexion at initial contact	10.8
KSTS5^a^ - Increased flexion in midstance and internal flexion or extension moment present	31.8
**Knee during swing**	
KSWS0—Normal knee motion during swing–no or minor gait deviations	35.4
KSWS1—Delayed peak knee flexion	21.5
KSWS2—Increased peak knee flexion	12.6
KSWS3—Increased and delayed peak knee flexion	9.4
KSWS4—Decreased peak knee flexion	10.8
KSWS5—Decreased and delayed peak knee flexion	10.2
**Ankle during stance**	
ASTS0—Normal ankle motion during stance–no or minor gait deviations	38.6
ASTS1—Horizontal second ankle rocker	27.9
ASTS2—Reversed second ankle rocker	9.4
ASTS3—Equinus gait	4.2
ASTS4—Calcaneus gait	19.8
**Ankle during swing**	
ASWS0—Normal ankle motion during swing–no or minor gait deviations	40.0
ASWS1—Insufficient prepositioning in terminal swing	6.7
ASWS2—Continuous plantarflexion during swing (drop foot)	18.6
ASWS3—Excessive dorsiflexion during swing	34.7
**CORONAL PLANE**	
**Pelvis**
PC0—Normal pelvic motion/posture–no or minor gait deviations	48.5
PC1—Increased pelvic range of motion	29.1
PC2—Continuous pelvic elevation	11.8
PC3—Continuous pelvic depression	10.6
**Hip**	
HC0—Normal hip motion–no or minor gait deviations	62.8
HC1—Excessive hip abduction in swing	21.6
HC2—Continuous excessive hip abduction	9.2
HC3—Continuous excessive hip adduction	6.5
**TRANSVERSE PLANE**	
**Pelvis**	
PT0—Normal pelvic motion/posture–no or minor gait deviations	44.5
PT1—Increased pelvic range of motion	30.3
PT2—Excessive pelvic external rotation during the gait cycle	13.0
PT3—Excessive pelvic internal rotation during the gait cycle	12.2
**Hip**	
HT0—Normal hip motion–no or minor gait deviations	75.3
HT1—Excessive hip external rotation during the gait cycle	9.0
HT2—Excessive hip internal rotation during the gait cycle	15.7
**Foot**	
FPA0—Normal foot progression angle–no or minor gait deviations	66.4
FPA1 –Out-toeing	15.6
FPA2 –In-toeing		17.9

Observed frequency (%) and brief description of all sagittal, coronal, and transverse plane joint motion patterns as defined by the experts in the Delphi-consensus study of Nieuwenhuys et al. [6]. Described deviations such as increased or excessive joint angles refer to deviations that are clearly deviating from the reference database of children developing normally, according to the detailed description that is available in [6].

^a^ The knee joint patterns KSTS5 and KSTS6 from [6] were merged as they only differ in the kinetics while this study focused on the kinematic features.

**Table 2:** Expert-defined features and discretization.

	Expert features	Expert discretization	Number of bins
ASTS	SRA	[-21,-5.5,19.4,31]	3
	aMaxStSagA	[-40,0,20,38]	3
ASWS	aIc2SagA	[-39,-4.2,80]	2
	aSagA-pct-GC-900	[-42,-2,31]	2
	aBelow1SDSwSagApct	[0,50,100]	2
	aAbove1SDSwSagApct	[0,33,100]	2
KSTS	aIcSagK	[-17,13.6,77]	2
	pctaMaxMStSagK	[0,11.2,30]	2
	aMinStSagK	[-33,-3.8,7.9,70]	3
KSWS	aMaxSwSagK	[5,54.4,67,98]]	3
	DeFlKpctSw	[1.5,35.6,99]	2
PS	ARomSagP	[1,5.4,23]	2
	PS-f2	[aAbove1SDSagP,aBelow1SDSagP]	3
HS	aMinStSagH	[-32,-4.3,40]	2
	aRomStSagH	[8,38.3,73]	2
	aAbove1SDSagHpct	[0,90,100]	2
PC	aRomCorP	[1,12.8,26]	2
	PC-f2	[aAbove1SDCorP,aBelow1SDCorP]	3
HC	aBelow1SDSwCorHpct	[0,50,100]	2
	HC-f2	[aAbove1SDCorHpct,aBelow1SDCorHpct]	3
PT	aRomTransP	[0,18,53]	2
	PT-f2	[aAbove1SDTransP,aBelow1SDTransP]	3
HT	HT-f1	[aAbove1SDTransH,aBelow1SDTransH]	3
FT	FT-f1	[aAbove1SDStTransF,aBelow1SDStTransF]	3

The expert-defined discretization for the kinematic features from [6]. Two examples for interpreting the discretization: (1) for the SRA feature of ASTS the resulting bins are: bin1 = [-21,-5.5); bin2 = [-5.5,19.4), bin3 = [19.4,31]; and (2) for the PS-f2 feature of PS: bin1 = [aAbove1SDSagP = true], bin2 = [aBelow1SDSagP = true], bin3 = [aAbove1SDSagP = false, aBelow1SDSagP = false] (co-occurrence is physically impossible).

In Van Gestel et al.’s [8] study there are more setting similarities with the current study, and Bayesian networks were used for probabilistic classification. However, in addition to testing a Bayesian network classifier this study also applied Logistic Regression. Moreover, we used the most recent consensus-based joint motion patterns [6] as available expert knowledge, which have evolved considerably since the study of Van Gestel et al. [8]. Van Gestel et al. [8] only applied the classification to four joint motions (sagittal knee and ankle motion in stance and swing), while this paper reports on results for eleven joint motions (Table 1). The studied population in the current study was also larger (356 patients versus 139 patients) and we explored different approaches to incorporate expert knowledge (feature selection and discretization), which have not been studied before in the field of CP gait pathology.

By developing a quantitative classification approach that uses joint motion patterns and gait features defined and discretized by clinical experts as its essential characteristics, the clinical relevance of the automatic classification and its clinical acceptance will improve. This study states two hypotheses to evaluate automatic probabilistic joint motion gait classification in children with CP incorporating the newly available expert knowledge from the consensus-based classification [6]:

1. Joint motion patterns in children with CP, obtained through a Delphi-consensus study, can be automatically classified following a probabilistic approach, with an accuracy similar to clinical expert classification.
2. The inclusion of clinical expert knowledge in the selection of relevant gait features (hypothesis 2a) and in the discretization of continuous features (hypothesis 2b), increases the performance of automatic probabilistic joint motion classification.

## Materials and methods

### Patient group

After the project was approved by the Medical Ethics Committee of UZ Leuven (Leuven University Hospitals) (ref. s56036), the clinical motion analysis laboratory’s database of Pellenberg University Hospital was searched for gait analysis sessions of children with unilateral or bilateral spastic CP, aged between 3 to 18 years and GMFCS levels I, II, or III. Children with marked signs of dystonia or ataxia were excluded, but any previous treatments were allowed. All patient information was anonymized prior to statistical analysis.

### Data collection

The method of data collection corresponds with Nieuwenhuys et al.’s [9] methodology. Briefly, the data was obtained by a standardized 3DGA measurement using optoelectronic cameras (Vicon Motion Systems, Oxford, UK), observing reflective markers, attached by clinical experts to the anatomical landmarks of the child’s lower legs, following the Plug-In-Gait marker configuration. Children were walking barefoot at a self-selected speed. The joint angles and their derivatives were obtained through the Nexus software. Additionally, the kinematic data was time-normalized to the overall gait cycle (pelvis in the sagittal (PS), coronal (PC), and transverse (PT) plane; hip in the sagittal (HS), coronal (HC), and transverse (HT) plane; and foot progression angle (FPA)) or to the stance and swing phase (knee during stance (KSTS) and during swing (KSWS) in the sagittal plane; ankle during stance (ASTS) and during swing (ASWS) in the sagittal plane) and interpolated resulting in 51 data points for each time-varying variable. All available trials were included in the study and classified according to the consensus-based joint motion patterns [6], by one of the two involved clinical experts. For each patient, 2 to 15 trials were used per session, with an average number of 4 trials. Multiple sessions per patient could be included, involving sessions before and after intervention (botulinum toxin injections, selective dorsal rhizotomy or orthopaedic surgery), as well as follow-up sessions charting the natural evolution (275 patients had one session, 67 had two sessions and 14 patients had more than two sessions included in the database). The interval between gait analysis sessions was 2 to 3 months for botulinum toxin injections, and one year for selective dorsal rhizotomy and orthopaedic surgery. Including gait analysis sessions before and after interventions created a generic database that facilitates the development of classification algorithms that are valid for all common clinically patient conditions. This classification was used as the ground-truth when training and validating the automatic classifiers. Pathological gait patterns were classified comparing average walking patterns of 56 children, aged between 5 and 18 years, who display normal development and had no previous history of neuromotor or musculoskeletal disorders. Table 1 presents a brief description of the consensus-based joint motion patterns and their observed frequency in the data set of this study. For the development of the algorithms, the knee joint motion patterns KSTS5 and KSTS6 from [6] were merged as the only difference between these two joint motion patterns is in the kinetic features, which were not considered in this study.

In total, the dataset consists of 356 patients and 1,719 gait trials. Moreover, 1,010 features were identified including the interpolated joint motion measurements and the discrete features extracted from these measurements.

### Automatic probabilistic classification algorithms

The goal of the automatic probabilistic classification was to classify the eleven different joint motions occurring in the gait of a child with CP as one of the classes (joint motion patterns) defined by experts (Table 1). On top of this, rather than providing a hard assignment, the output of the probabilistic classification equals the probabilities that the child’s joint motions belong to one of the clinically relevant joint motion patterns. To this end, this study used a supervised learning approach that uses joint motions classified by experts to train the algorithms. To investigate how expert knowledge can improve automatic probabilistic gait classification, this study applied four approaches, each using the available expert knowledge to a different extent. In the first approach *(approach 1)*, the expert knowledge was used maximally, i.e. the classification rules include the expert-defined features and the discretization rules of the continuous features (Table 2). In the second approach *(approach 2)* all available 1,010 features were fed into to the classification algorithm. A naïve approach that inputs all the features directly into the classification algorithm *(approach 2a)* was compared with an approach where data-driven feature selection precedes the classification *(approach 2b)*. Finally, the third approach *(approach 3)* did not use the discretization of continuous features as defined by the expert, but rather used the continuous features directly, or learned the discretization rules from the data.

The exploration of the effect of different levels of expert knowledge is of interest for four reasons. Firstly, an approach that maximally uses the expert knowledge is expected to demonstrate improved compliance with the expert-based classification and therefore expected to obtain higher classification performance. However, this is only be the case if the expert-defined classification rules (relevant features and the discretization of these features) supply the underlying the expert rationale. If experts use knowledge that is not contained in the classification rules (for example by focusing on the overall gait pattern instead of the features they identified as important), the emerging automatic classification will obtain lower classification performance when compared to experts. Therefore, a high classification performance of the first approach, which uses expert knowledge to a maximum degree, thus works to confirm the validity of the expert-defined classification rules. Secondly, the process of creating an expert-defined classification and classification rules is labour intensive (in this case a Delphi-based consensus classification was used) and depends on the experts involved in the process (subjectivity). While on the other hand, an automatic data-driven feature selection and discretization process (approach 2 and 3) is considered to be objective. Moreover, by using data-driven feature selection, less obvious features and discretization could potentially be detected, resulting in an even higher classification performance than was obtained by using full-expert knowledge (approach 1). Thirdly, while automatic feature selection and discretization is objective, the selected features and identified discretization rules will depend on the database used for learning. As developing gait database that correctly captures a wide population is far from trivial, there is a risk of overfitting to the available gait data. Fourthly, using automatic feature selection and discretization comes at a higher computational cost and bears the risk of decreasing clinical interpretability. Automatic procedures might select features that, according to experts, are not directly related to the gait patterns or might discretize features into bins that do not have any clinical meaning.

## Theory

### Evaluation and performance measures

To evaluate the performance of the classification approaches, stratified ten-fold cross validation was used. The folds were constructed manually such that multiple trials of a single patient were all placed in the same fold and such that different joint motion patterns were evenly distributed across the different folds (stratified sampling). The same folds were used to evaluate each of the approaches presented in this study.

Classification performance was measured by accuracy and f-score. Accuracy was expressed as the percentage of correctly classified trials. The joint motion patterns assigned by the two clinical experts was taken as the ground-truth. Accuracy might be misleading in the case of unbalanced (skewed) classes. As the posed classification problem was skewed (Table 1), the f-score, which combines the more robust precision and recall in a single measure, was additionally reported.

To assess the performance of the multi-class classification, macro-averaging was used as it treats all classes equally, independent of class size. This makes macro-averaging the preferred measure when high performance for small classes is desired. Macro-averaging defines the overall classification accuracy and f-score as the average across the different classes [12].

### Naïve Bayes (NB) and Logistic Regression (LR)

This study compared the performance for the four proposed classification approaches for two different classification algorithms: Naïve Bayes (NB) and Logistic Regression (LR).

#### Naïve Bayes

**Fig 1:**
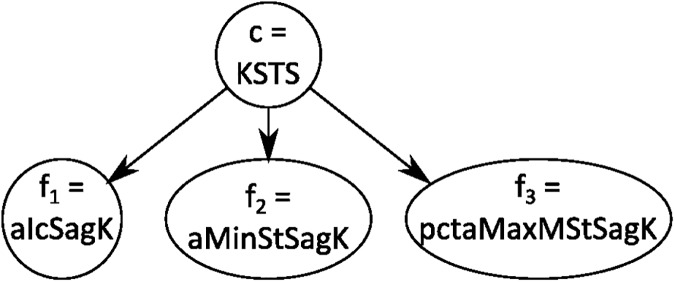
Bayesian network for naïve Bayes classifier. Example of Bayesian Network for naïve Bayes classifier for the knee in stance in the sagittal plane (KSTS). The parent random variable is the joint motion KSTS, which can take states {KSTS0, KSTS1, KSTS2, KSTS3, KSTS4, KSTS5} (the expert-defined joint motion patterns, Table **1**). The child random variables are the expert-defined features (Table 2). For KSTS there are three expert-defined features: alcSagK, aMinStSagK, pctaMaxMstSagK. The arrows depict the probabilistic relationship between the parent and child node, in this case: the probability that a feature has a particular value, given the joint motion pattern: e.g. *p*(*f*_1_ = 0|*KSTS* = *KSTS*1).

A naïve Bayes (NB) classifier simplifies the classification problem by assuming that the observed features are independent of each other, given the class to which the pattern belongs. The Bayesian network underlying the classifier graphically illustrates this independency. Fig 1 shows the Bayesian network for the naïve Bayes classifier of the knee in stance in the sagittal plane (KSTS). The parent random variable is the joint motion pattern (*c*_*j*_) and the child random variables (*f*_1_, *f*_2_, …, *f*_*n*_) are the features from the 3DGA. To fully specify the BN the conditional probability tables (CPTs) *p*(*f*_*i*_|*c*_*j*_) of each of the features given the joint motion patterns should be specified or learned. In this study, the maximum likelihood estimates of the CPTs were learned from the available expert classification by simple counting [13]. Once these are available, the posterior probability of a patient’s joint motion belonging to a particular joint motion pattern *c*_*j*_ given the observed features (*f*_1_, *f*_2_, …, *f*_*n*_) was calculated as:

$$p(c_{j}|f_{1},f_{2},\ldots ,f_{n})=\frac{\prod_{i=1}^{n}p(f_{i}|c_{j})p(c_{j})}{p(f_{1},f_{2},\ldots ,f_{n})},$$ (1)

where *p*(*f*_1_, *f*_2_, …, *f*_*n*_) is a mere normalisation constant. When performing hard assignments, the joint motion was assigned to the maximum posterior probability joint motion pattern, i.e.:

$$c_{MAP}={\mathrm{argmax}}_{c_{1},\ldots ,c_{m}}p(c_{j}|f_{1},f_{2},\ldots ,f_{n})=\underset{c_{1},\ldots ,c_{m}}{argmax}\;\prod_{i=1}^{n}p(f_{i}|c_{j})p(c_{j}).$$ (2)

#### Logistic regression

Logistic regression (here equations are provided for binary classification, for multinomial classification we refer to [14], chapter 8) maps the input, in this case the features (*f*_1_, *f*_2_, …, *f*_*n*_) to the output, in this case to the probability of belonging to the joint motion pattern *c*_*j*_.

$${p(c_{j}|f_{1},f_{2},\ldots ,f_{n})=Ber(h}_{\theta }(f_{1},f_{2},\ldots ,f_{n}))=Ber\left(\frac{1}{1+e^{-(\theta_{0}+\sum_{i=1}^{n}\theta_{i}f_{i})}}\right).$$ (3)

Supervised maximum-likelihood learning consists of finding the parameters *θ* that fit the training data optimally while not overfitting. To this end a cost function *J*(*θ*) that measures the misfit between the predicted probability of belonging to the different joint motion patterns and the ground-truth joint motion pattern *c*_*l*_ of the K training instances while adding a regularization (*λ*) to penalize overfitting, is minimized:

$$\begin{array}{l} \theta_{MAP}={\mathrm{argmin}}_{\theta }J(\theta ),\;\mathrm{with}\\ J(\theta )=\sum_{k=1}^{K}[-c_{l}^{\left(k\right)}\;\log\left(h_{\theta }(f_{1}^{\left(k\right)},f_{2}^{\left(k\right)},\ldots ,f_{n}^{\left(k\right)})\right)-(1-c_{l}^{\left(k\right)})\;\log\left(1-h_{\theta }(f_{1}^{\left(k\right)},f_{2}^{\left(k\right)},\ldots ,f_{n}^{\left(k\right)})\right)]+\lambda \sum_{i=1}^{n}\theta_{i}. \end{array}$$ (4)

As the cost function is convex, there are no local minima and minimization can be carried out using standard optimization algorithms such as gradient descent or Newton’s method, as employed in this study.

### Data-driven feature selection

The goal of data-driven feature selection (FS) is to reduce the feature subset while trying to maintain the information present in the original feature set. FS removes irrelevant data, while often still increasing the predictive accuracy of the learned model and thus reduces the computational complexity and increases the learning efficiency. Moreover, by using a reduced feature set, the interpretability can be increased [15,16]. This study used correlation-based feature selection, which aims at selecting a subset of features that individually correlate well with the class but have little inter-correlation [17]. Moreover, a best-first search heuristic [17] was used and the search was terminated when five consecutive non-improving iterations occurred. The resulting subset was used as input for the NB and LR classifiers.

### Automatic feature discretization

For NB, entropy-based discretization [18,19] was used to discretize the features selected by experts, which is reported to improve classification performance [19]. As the LR classifier can easily handle continuous features, they were directly fed into the algorithm.

## Results

### Hypothesis 1: Joint motion patterns in children with CP, obtained through a Delphi-consensus study, can be automatically classified following a probabilistic approach, with an accuracy similar to clinical expert classification

Table 3 provides the results for all joint motions of the NB and LR classifiers using approach 1 (abbreviated as NB1 and LR1), i.e. when using the expert-defined features and discretization. The overall accuracy and f-score of both NB1 and LR1 were 91% and 90%, respectively. For the different joint motion patterns, the accuracy and f-score range from 75% and 72% (KSTS) to 98% and 96% (HT) respectively. The overall performance of NB1 and LR1 were similar, with small variations for the different joint motion patterns.

Nieuwenhuys et al. [9] reported the level of agreement with which clinicians could recognize specific joint motion gait patterns in children with cerebral palsy (CP) as defined by the consensus study [20]. Therefore, to compare the performance of the automatic probabilistic joint motion classification with respect to clinical expert classification, the accuracy and f-score of NB and LR proposed in this study were compared to the Percentage-Of-Agreement (POA) reported by Nieuwenhuys et al. [9]. The POA are also available in Table 3. The overall accuracy of both NB1 and LR1 (91%) was higher than both the inter-rater POA of a group of 28 raters (RG1, 78%) and of two expert raters of the research team developing the classification (RG2, 90%). In addition, the accuracy of both NB1 and LR1 was higher than the POA of RG1 for all joint motion patterns. For RG2, the POA was higher than the accuracy of NB1 and LR1 for four joint motion patterns (NB1: ASWS, HS, PC, FT; LR1: KSWS, HS, PC, FT). Only for the hip in the sagittal plane (HS) was this difference higher than 3% and reached a significant value of 9% and 10% for NB1 and LR1 respectively.

**Table 3:** Performance of automatic classification using expert-defined and discretized features.

	NB1		LR1		Inter-rater POA [9]	
	accuracy	f-score	accuracy	f-score	RG1	RG2
ASTS	90	91	89	90	76	88
ASWS	86	85	89	86	74	87
KSTS	75	72	75	72	58	68
KSWS	90	89	89	89	77	90
PS	92	90	92	92	77	85
HS	84	83	85	83	78	94
PC	97	97	97	97	79	98
HC	92	92	92	92	78	91
PT	96	97	96	96	79	99
HT	98	96	98	96	87	96
FT	97	96	97	96	91	95
**overall**	**91**	**90**	**91**	**90**	**78**	**90**

Performance, expressed in percentages, of NB and LR for classification using expert-defined features and discretization compared with level of agreement by clinicians, expressed as percentage of agreement (POA) as reported in [9] for a group of 28 trained raters with clinical background (RG1) and two expert raters (RG2). For each joint motion, the accuracy and f-score of the algorithm with highest performance are indicated in grey.

**Fig 2:**
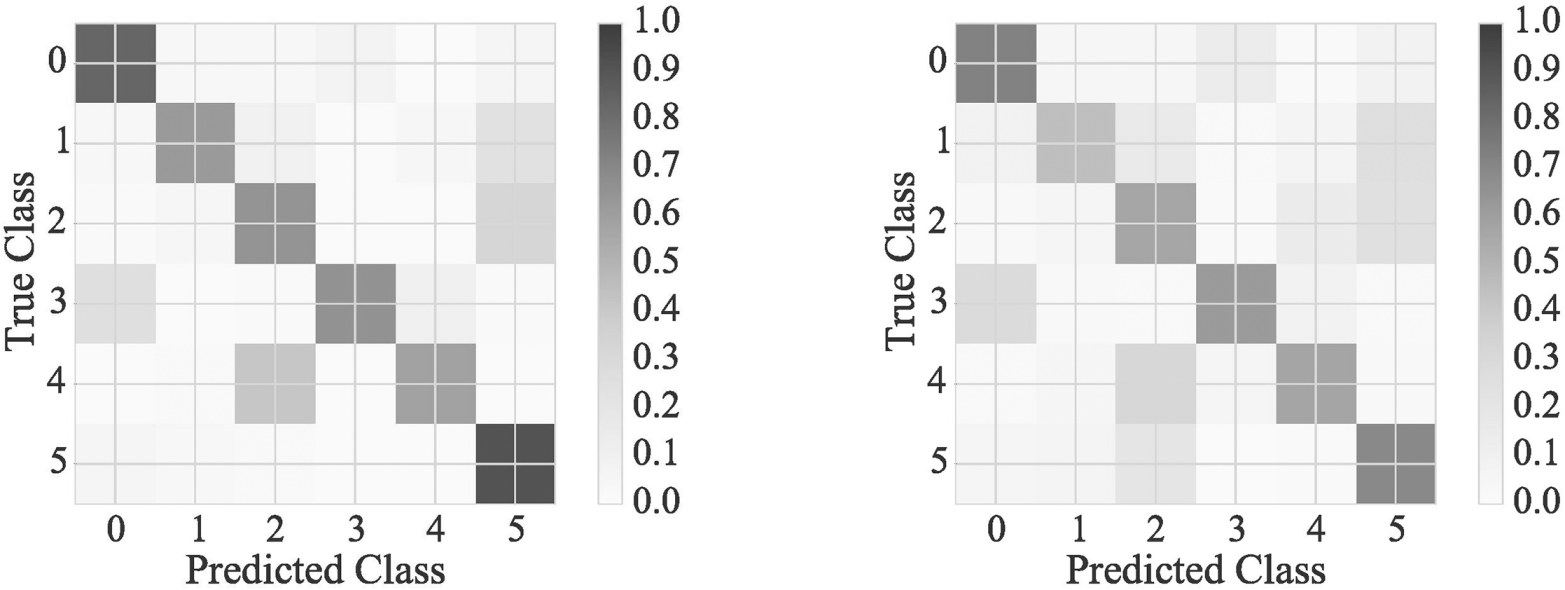
Classification performance details for Naïve Bayes and KSTS. Normalized confusion matrix (left) and average posterior probability matrix (right) for KSTS obtained by NB using expert-defined features (NB1). Each entry (i,j) in the confusion matrix shows the fraction of all joint motions that according to the expert belong to joint motion pattern i (True Class) are actually classified as joint motion pattern j (Predicted Class). Each entry (i,j) in the average posterior probability matrix shows the average posterior probability of all joint motions that according to the expert belong to joint motion pattern i (True Class) originating from joint motion pattern j (Predicted Class) according to the classifier. So the entry (i,j) of the average posterior probability matrix contains the average of *p*(*c*_*j*_|*f*_1_, *f*_2_, …, *f*_*n*_) for all joint motions that belong to class i (True Class) according to the expert.

Confusion matrices and average posterior probabilities (Fig 2 for NB1 and KSTS, the S1 Appendix provides the results for all patterns and for both NB1 and LR1) provide more detail on the classification performance. The confusion matrix shows how the joint motions that belong to a particular joint motion pattern (True Class) according to the expert, are automatically classified into the different joint motion patterns (Predicted Class). Additionally, the average posterior probability matrix adds the information on the probability of the joint motions that according to the experts (True Class) belong to a particular joint motion pattern, originating from any other joint motion pattern (Predicted Class) according to the automatic classifier.

### Hypothesis 2: The inclusion of clinical expert knowledge increases classification performance

#### Hypothesis 2a: Gait feature selection

Table 4 shows the classification performance of NB and LR where all available features were used, both following the naïve approach (approach 2a, so NB2a and LR2a) as well as the approach with data-driven feature selection (approach 2b, so NB2b and LR2b). The S2 Appendix provides the detailed results using the confusion matrices and posterior probabilities for all patterns and for NB2a, LR2a, NB2b, and LR2b.

**Table 4:** Performance of classifiers using a naïve approach (all features) and data-driven feature selection.

	naïve approach				data-driven feature selection				
	NB2a		LR2a		NB2b		LR2b		number of features selected
	accuracy	f-score	accuracy	f-score	accuracy	f-score	accuracy	f-score	
ASTS	71	71	83	84	90	91	90	91	8
ASWS	74	69	69	64	87	85	88	87	14
KSTS	62	61	69	64	82	81	79	75	12
KSWS	68	66	82	75	92	92	92	91	11
PS	74	60	96	90	97	92	98	96	4
HS	82	80	86	86	89	90	89	89	7
PC	76	76	94	94	97	97	96	97	9
HC	86	80	89	89	94	94	94	94	9
PT	79	79	93	93	97	97	95	95	8
HT	90	86	96	94	97	96	98	96	9
FT	74	72	95	93	97	96	96	97	6
**overall**	**76**	**73**	**87**	**84**	**93**	**92**	**93**	**92**	

Performance, expressed in percentages, of NB and LR for classification using all features both for the naïve approach and the data-driven feature selection. For each joint motion, the accuracy and f-score of the algorithm with highest performance is indicated in grey.

The naïve approach (approach 2a) had lower performance (NB2a: 76% and 73%; LR2a: 87% and 84%) than the first approach which used the expert-defined features (Table 3; NB1 and LR1: 91% and 90%). For NB2a the accuracy for all joint motion patterns was lower than for NB1 (smallest difference for HS, 2%; largest difference for FT, 23%). For LR2a the accuracy for joint motion patterns PS and HS was slightly higher than for LR1 with accuracy improvements of 4% and 1%, respectively.

When using data-driven feature selection (approach 2b) the overall performance of the algorithms (NB2b and LR2b: 93% and 92%) was higher than when using the expert-defined features in NB1 and LR1 (accuracy + 2%; f-score +2%). However, the increase of performance was limited. For instance, when considering LR2b, for four (ASWS, PC, HT, FT) and three (PC, PT, HT) of the joint motion patterns, no increase in performance was observed with respect to LR1 when considering the accuracy and f-score, respectively.

Table 4 shows that the number of features obtained by data-driven feature selection was higher than the number of expert-defined features (Table 2). The data-driven feature selection selected on average 8.8 features per pattern while the experts only used on average 2.1 features to obtain similar performance.

#### Hypothesis 2b: Gait feature discretization

Table 5 shows the performance when only considering the features used by experts (approach 3). In NB3 the continuous features were automatically discretized while for LR3 the continuous features were used directly. The S3 Appendix provides the detailed results using the confusion matrices and posterior probabilities for all patterns and for both NB3 and LR3. LR3 with the continuous features outperformed NB3 with the learned discretization (accuracy +4%, f-score + 5%) and this was consistent for all joint motions. However, even for LR3 the performance (LR3: accuracy 90% and f-score 90%) was slightly lower overall than the algorithms using the expert-defined features (Table 3; LR1: accuracy 91% and f-score 90%). When considering the different joint motion patterns, the accuracy of NB3 was only higher than NB1 for one joint motion pattern (HS, +3%), and the accuracy of LR3 was only higher than LR1 for three joint motion patterns (KSTS, +2%; KSWS, +5%; HS, +3%).

**Table 5:** Performance of classifiers using automatically discretized features.

	NB3		LR3	
	accuracy	f-score	accuracy	f-score
ASTS	89	89	89	89
ASWS	66	68	77	78
KSTS	71	69	77	73
KSWS	89	89	94	93
PS	92	90	92	92
HS	87	86	88	87
PC	91	90	96	95
HC	85	81	92	92
PT	90	89	95	95
HT	90	87	97	95
FT	94	93	97	96
**overall**	**86**	**85**	**90**	**90**

Performance, expressed in percentages, of Naïve Bayes for classification using automatically discretized features (NB3) and Logistic Regression with continuous features (LR3). For each joint motion, the accuracy and f-score of the algorithm with highest performance is indicated in grey.

## Discussion

**Fig 3:**
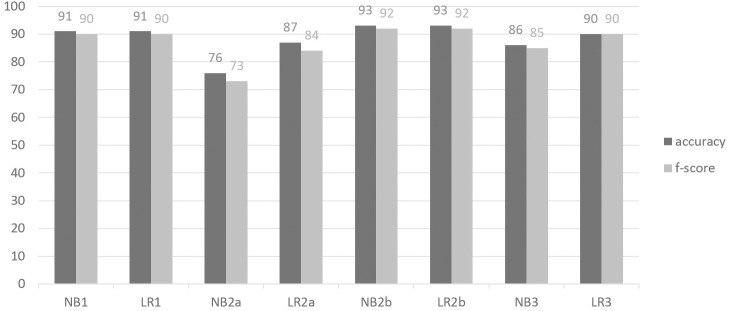
Summarized performance of different approaches. Performance, expressed in percentages, of the four approaches presented in this study. NB1 and LR1 represent the Naïve Bayes and Logistic Regression classifiers respectively using all expert-defined and discretized features (hypothesis 1, approach 1). NB2a and LR2a represent the Naïve Bayes and Logistic Regression classifiers respectively using all available features (hypothesis 2a, approach 2a). NB2b and LR2b represent the Naïve Bayes and Logistic Regression classifiers respectively using automatic feature selection (hypothesis 2a, approach 2b). NB3 and L3 represent the Naïve Bayes and Logistic Regression classifiers respectively using the expert-defined but automatically discretized (NB) or continuous (LR) features (hypothesis 2b, approach 3).

This study applied four different approaches, each using a different level of expert knowledge, to answer the two hypotheses put forward. Fig 3 summarizes the performance of the four approaches, each time for the two classifiers used: Naïve Bayes (NB) and Logistic Regression (LR).

The high performance of each of the approaches presented shows that the joint motion gait patterns in children with CP, obtained through a Delphi-consensus study, can be used for automatic probabilistic gait. Additionally, when exploiting all available expert knowledge, i.e. the expert-defined features and discretization rules, the overall accuracy of both NB1 and LR1 (91%) was higher than the inter-rater POA of two expert raters of the research team developing the classification (RG2, 90% [9]). This confirmed the first hypothesis and provides additional confidence that the consensus-based joint motion patterns are well-defined. Interestingly, the overall accuracy of the automatic classifiers was on average 13% higher than the POA of the group of more inexperienced raters (RG1, 78% [9]). This indicates that the automatic classification can be especially useful when supporting or training junior clinicians.

**Fig 4:**
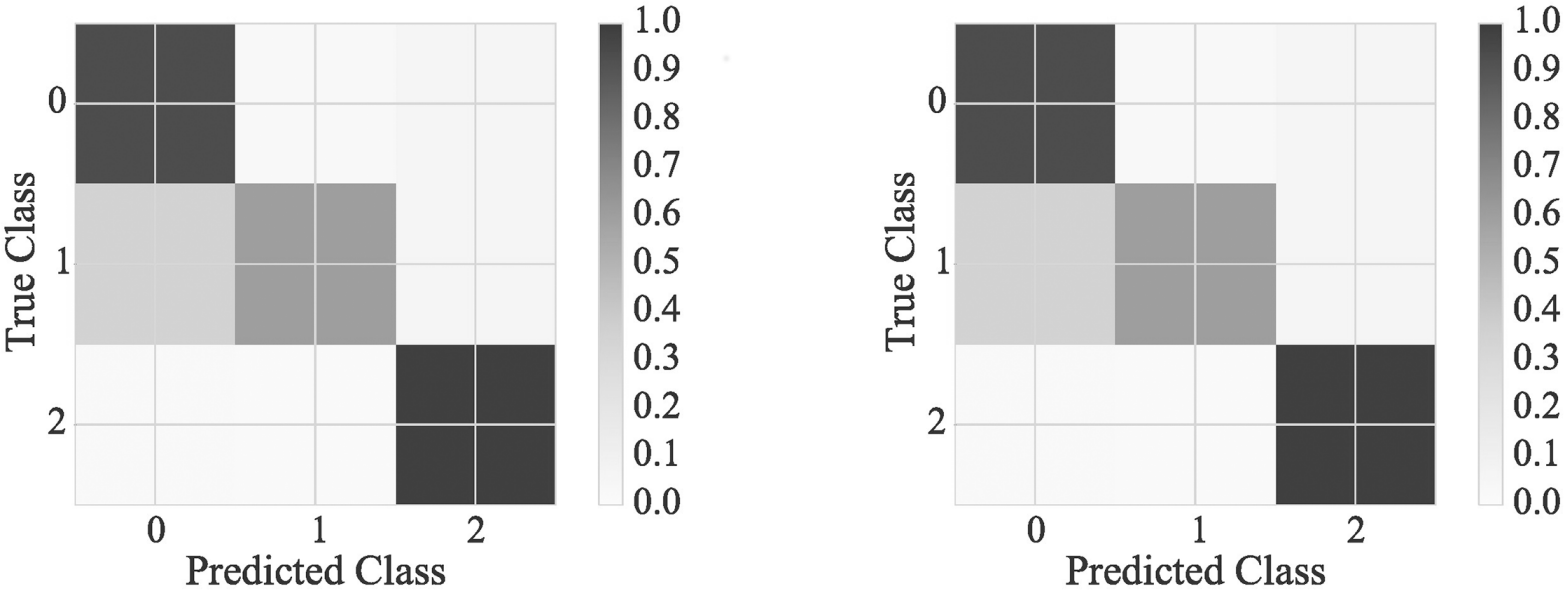
Normalized confusion matrix for Naïve Bayes and logistic regression using approach 1. Normalized confusion matrix for the hip in the sagittal plane (HS) for both Naïve Bayes (left, NB1) and Logistic Regression (right, LR1) classifiers using all expert-defined and discretized features (hypothesis 1, approach 1).

As detailed in the results section (Table 3), the accuracy of the automatic classification for the hip in the sagittal plane (HS) using expert-defined and discretized features (NB1, 84% and LR1, 85%) was significantly lower than the POA of the expert raters (RG2, 94% [9]), but higher than the non-expert raters (RG1, 78% [9]). The confusion matrices in Fig 4 indicate that the lower performance is caused by misclassification of joint motion patterns that are classified by the experts as HS1 (hip extension deficit) to HS0 (normal hip motion). While the use of the continuous feature (LR3) and automatic feature discretization (NB3) does improve accuracy (NB3, 87% and LR3, 88%), the accuracy is still a long ways away from expert rater accuracy. Therefore, this would suggesta reconsideration of the expert-defined features, and not only the discretization of the expert-defined features, for the hip in the sagittal plane. In particular, it should be checked whether the joint motion pattern definitions sufficiently capture the expert reasoning used when classifying this joint motion.

As an output, the probabilistic classification produces not only confusion matrices, but also posterior probabilities. In other words,. for each of the classified patients, or for the classified population as a whole, the probability of belonging to the different particular joint motion patterns is provided. As such, these posterior probabilities give feedback to clinicians as to which patterns can be potentially confused in an automatic classification. Clinicians may use this information to improve to the consensus-based patterns, as shown above for the HS joint motion patterns.

As already indicated in the literature [4–6], summarizing the vast amount of data obtained through 3DGA can facilitate clinical-decision making. This statement is supported by the results in this study. Firstly, when using a naïve classification approach using available features from the 3DGA (approach 2a), a lower performance (Table 4, NB2a: 76% and 73%; LR2a: 87% and 84%) than the algorithms using the expert-defined features (Table 3; NB1 and LR1: 91% and 90%) was obtained. The lower accuracy when naively using all available features was, however, alleviated by using data-driven feature selection: the overall performance of the algorithms (NB2b and LR2b: 93% and 92%) was slightly higher than when using the expert-defined features (accuracy + 2%; f-score +2%). However, this data-driven feature selection comes at a cost: additional computational time has to be allocated for the features’ selection; the number of features used to obtain similar classification performance is higher than the number of expert-defined features (Table 4, average of features per pattern 8.8 for data-driven feature selection and 2.1 for expert-defined features); the clinical interpretability of the selected features is not guaranteed. Therefore, taking Occam’s razorand the importance of clinical interpretability for clinical acceptance into account, we instead recommend using automatic feature selection as feedback to experts to help them optimize joint motion pattern definitions, rather than as a basis for classification. Secondly, when using the expert-defined features, rather than the continuous version (LR3) or automatic feature discretization (NB3), the accuracy of the classification decreases (LR -1%, NB -5%). The use of these continuous features or the automatic discretization of the features also brings an additional computational cost. Moreover, as with the data-driven feature selection, the automatic feature discretization might produce discretized features which are hard to interpret clinically. Therefore, we recommend using expert-defined and discretized features rather than continuous or automatically discretized features. Based on the discussion above we can conclude that hypothesis 2 does not hold. Automatic feature selection and discretization can result in slightly higher joint motion classification performance. However, the increase in performance is limited and does not outweigh the additional computational cost and issues of clinical acceptance and applicability.

When comparing the classification performance to the earlier application of Bayesian networks using clinical expert-knowledge by Van Gestel et al. [8], this study (NB1) consistently obtained a higher accuracy for the common joint motion patterns (ASTS +2%, ASWS +3%, KSTS +1%, KSWS +3%) using a larger database (+217 patients). As the approach of Van Gestel et al. [8] was the same as the Naïve Bayes in approach 1 in this study (NB1), the increase in performance can only be explained by the improved pattern definitions in the consensus study [6]. Interestingly, the consensus study used the insight gained from the automatic joint motion classification of Van Gestel et al. [8]. Therefore, the new results reported in this study can again be used to improve the pattern definitions of the consensus study.

A limitation of the paper is that the results are based on the gait database of the Clinical Motion Analysis Laboratory of UZ Leuven. While the gait database is expansive, and includes a large variety of patients, there is no guarantee that similar results would be obtained with other gait pattern databases from other clinical centres. Moreover, the available reference database defined the threshold values used in the definition of the expert-defined features, and may thus have an impact on the classifications created by experts and therefore also on the automatic classification. The inclusion of multiple trials of the same patient is another point worth discussing. The decision to include multiple trials of the same patient or not is a balance between including all available knowledge and hereby maximizing the size and generalizability of the database and the risk of duplicating information (and hereby attaching too much weight to patients with more trials). On the other hand, often different trials of the same patient differ to a large extent and therefore including multiple trials of the same patient might help to capture the variability and even inconsistency that exists for a single patient. As having a large database that captures this variability was considered important, this paper includes all available trials. However, as using gait trials of the same patient in the training and test might result in overestimating the classification performance, the training and test sets were constructed with great care. As all trials of the same patients were placed in the same fold, the 10-fold cross validation never used data of the same patient in both the training and the test set. Therefore, the toughest classification setting possible was constructed.

A second limitation of the paper is that the expert knowledge and expert classification underlying this paper solely relies on the consensus-based gait pattern classification of Nieuwenhuys et al. [6]. The application of the same methodology to other gait pattern classifications is the subject of future study.

The performance reported in this study (accuracy and f-score) was based on the hard assignment to the joint motion pattern with highest posterior probability. The probabilistic classification, however, provided the probability of belonging to any of the joint motion patterns, therefore opening the way to a soft assignment. As shown by Van Gestel et al. [8], accuracy measures based on hard assignment likely underestimate the true performance, i.e. performance measured by incorporating the soft assignment in the accuracy measure. Therefore, for future work, accuracy measures taking into account the soft assignment and benchmarking it to the doubt indicated by experts in the reliability study [9], should be taken into consideration. Additionally, the clinical added value of the use of the posterior probabilities as “confidence” in the automatic classification should be explored further. The use of the posterior probabilities can be especially useful when classifying a single new joint motion as it can trigger the clinicians to appropriately interpret the automatic classification, or to make adaptations where necessary.

## Conclusion

This study developed algorithms for the automatic probabilistic joint motion gait classification in children with CP by using the newly available expert knowledge from the consensus-based classification [6]. To this end the study applied a Bayesian network and Logistic Regression in four different approaches, each with a different level of use of expert knowledge (expert-defined and discretized features). Firstly, the results showed that the joint motion patterns, obtained through the Delphi-consensus study [6], can be used to automatically classify joint motions of children with CP following a probabilistic approach, with an accuracy similar to clinical expert classification. Furthermore, it was shown that the automatic classification obtains higher performance than non-experts. As such, the automatic classification has potential for supporting clinicians and medical practitioners in their clinical reasoning and decision making, supporting or training junior clinicians, as well as facilitating and enabling standardize use of this classification system among clinicians. In general, the automatic classification supports the purposes of classification of CP is nicely outlined by Bax et al. [21]: description, prediction, comparison, and evaluation of change. Nieuwenhuys elaborates on the potential uses of consensus-based classification of gait in children with CP in [22] Secondly, the results showed that the use of more advanced machine learning techniques such as automatic feature selection and discretization, instead of the expert-defined and discretized features can result in slightly higher joint motion classification performance. However, the increase of performance is limited and does not outweigh the additional computational cost and the higher risk of losing clinical interpretability, which threatens clinical acceptance and applicability. Therefore, we conclude that an automatic probabilistic classification that maximally uses the available expert-knowledge from the consensus-based classification [6] is preferred. Future works should concentrate on showing the clinical relevance and applicability of the automatic probabilistic joint motion classification in a clinical context, as well as the possibility of transferring this knowledge to other gait laboratories.

## Supporting information

S1 Appendix Classification results for hypothesis 1 (NB1 and LR1). (PDF)

S2 Appendix Classification results for hypothesis 2a (NB2a, LR2a, NB2b, LR2b). (PDF)

S3 Appendix Classification results for hypothesis 2b (NB3, LR3). (PDF)

## References

[1] Oskoui M, Coutinho F, Dykeman J, Jetté N, Pringsheim T. An update on the prevalence of cerebral palsy: a systematic review and meta-analysis. Dev Med Child Neurol. 2013;55: 509–519. doi: 10.1111/dmcn.12080 23346889 10.1111/dmcn.12080

[2] Dobson F, Morris ME, Baker R, Graham HK. Gait classification in children with cerebral palsy: a systematic review. Gait Posture. 2007;25: 140–52. doi: 10.1016/j.gaitpost.2006.01.003 16490354 10.1016/j.gaitpost.2006.01.003

[3] Chau T. A review of analytical techniques for gait data. Part 1: Fuzzy, statistical and fractal methods. Gait and Posture. 2001 pp. 49–66. 11166554 10.1016/s0966-6362(00)00094-1

[4] Wren TAL, Otsuka NY, Bowen RE, Scaduto AA, Chan LS, Sheng M, et al Influence of gait analysis on decision-making for lower extremity orthopaedic surgery: Baseline data from a randomized controlled trial. Gait Posture. 2011;34: 364–9. doi: 10.1016/j.gaitpost.2011.06.002 21723131 10.1016/j.gaitpost.2011.06.002

[5] Lofterød B, Terjesen T. Results of treatment when orthopaedic surgeons follow gait-analysis recommendations in children with CP. Dev Med Child Neurol. 2008;50: 503–509. doi: 10.1111/j.1469-8749.2008.03018.x 18611199 10.1111/j.1469-8749.2008.03018.x

[6] Nieuwenhuys A, Õunpuu S, Van Campenhout A, Theologis T, De Cat J, Stout J, et al Identification of joint patterns during gait in children with cerebral palsy: A Delphi consensus study. Dev Med Child Neurol. 2016;58: 306–313. doi: 10.1111/dmcn.12892 26330338 10.1111/dmcn.12892

[7] Rodda JM, Graham HK, Carson L, Galea MP, Wolfe R. Sagittal gait patterns in spastic diplegia. J Bone Joint Surg Br. 2004;86: 251–258. 15046442 10.1302/0301-620x.86b2.13878

[8] Van Gestel L, De Laet T, Di Lello E, Bruyninckx H, Molenaers G, Van Campenhout A, et al Probabilistic gait classification in children with cerebral palsy: A Bayesian approach. Res Dev Disabil. 2011;32: 2542–2552. doi: 10.1016/j.ridd.2011.07.004 21807478 10.1016/j.ridd.2011.07.004

[9] Nieuwenhuys A, Papageorgiou E, Molenaers G, Monari D, De Laet T, Desloovere K. Inter- and intrarater clinician agreement on joint motion patterns during gait in children with cerebral palsy. Dev Med Child Neurol. 2017; 10.1111/dmcn.13404 28224608

[10] Nieuwenhuys A, Papageorgiou E, Desloovere K, Molenaers G, De Laet T. Statistical Parametric Mapping to Identify Differences between Consensus-based Joint Patterns during Gait in Children with Cerebral Palsy. PLoS One. 2017; 10.1371/journal.pone.0169834 PMC5231378 28081229

[11] Zhang B ling, Zhang Y, Begg RK. Gait classification in children with cerebral palsy by Bayesian approach. Pattern Recognit. 2009;42: 581–586.

[12] Sokolova M, Lapalme G. A systematic analysis of performance measures for classification tasks. Inf Process Manag. 2009;45: 427–437.

[13] Koller D, Friedman N. Probabilistic Graphical Models: Principles and Techniques (Adaptive Computation and Machine Learning series). Foundations. The MIT Press; 2009.

[14] P. Murphy K. Machine Learning: A Probabilistic Perspective [Internet]. Machine Learning: A Probabilistic Perspective. 2012.

[15] García, SalvadorLuengo J, Herrera F, García S, Luengo J, Herrera F. Data Preprocessing in Data Mining [Internet]. Intelligent Systems Reference Library. 2015.

[16] Saeys Y, Inza I, Larrañaga P. A review of feature selection techniques in bioinformatics. Bioinformatics. 2007 pp. 2507–2517. doi: 10.1093/bioinformatics/btm344 17720704 10.1093/bioinformatics/btm344

[17] Witten IH, Frank E, Hall M a. Data Mining: Practical Machine Learning Tools and Techniques (Google eBook) [Internet]. Complementary literature None. 2011.

[18] Fayyad UM, Irani KB. Multi-Interval Discretization of Continuos-Valued Attributes for Classification Learning. Proceedings of the International Joint Conference on Uncertainty in AI. 1993. pp. 1022–1027. Available: http://trs-new.jpl.nasa.gov/dspace/handle/2014/35171

[19] Dougherty J, Kohavi R, Sahami M. Supervised and unsupervised discretization of continuous features. Mach Learn Proc Twelfth Int Conf. 1995;54: 194–202.

[20] Nieuwenhuys A, Õunpuu S, Van Campenhout A, Theologis T, De Cat J, Stout J, et al Identification of joint patterns during gait in children with cerebral palsy: A Delphi consensus study. Dev Med Child Neurol. 2016;58: 306–313. doi: 10.1111/dmcn.12892 26330338 10.1111/dmcn.12892

[21] Bax M, Goldstein M, Rosenbaum P, Leviton A, Paneth N, Dan B, et al Proposed definition and classification of cerebral palsy, April 2005. Dev Med Child Neurol. 2005;47: 571–6. Available: http://www.ncbi.nlm.nih.gov/pubmed/16108461 16108461 10.1017/s001216220500112x

[22] Nieuwenhuys A, Desloovere K, Molenaers G, De Laet T. Consensus-based classification of gait in children with cerebral palsy [Internet]. KU Leuven. 2016. Available: https://lirias.kuleuven.be/handle/123456789/551106

