# Supplementary material for: Does expert knowledge improve automatic probabilistic classification of gait joint motion patterns in children with cerebral palsy?
Source: PLoS One. 2017 Jun 1;12(6):e0178378. doi: 10.1371/journal.pone.0178378 (PMC5453476; doi:10.1371/journal.pone.0178378)
Supplement: S2 Appendix — (PDF) [file pone.0178378.s002.pdf]

Supplemental Material 2: Classification results for hypothesis 2a

This supplemental material provides the normalized confusion matrices and posterior probabilities for all classification tasks performed using Naive Bayes (NB) and Logistic Regression (LR) when using all features (NB2a in Fig.1, LR2a in Fig.2) and when using automatic feature detection (NB2b in Fig.3, LR2b in Fig.4) .

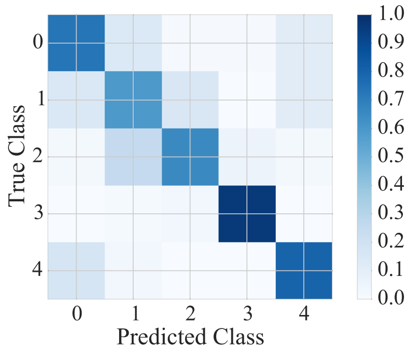

(a) $ASTS$ normalized confusion matrix

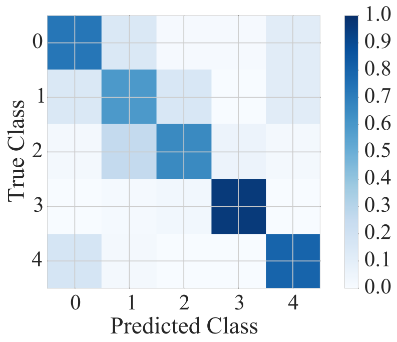

(b) $ASTS$ posterior probabilities

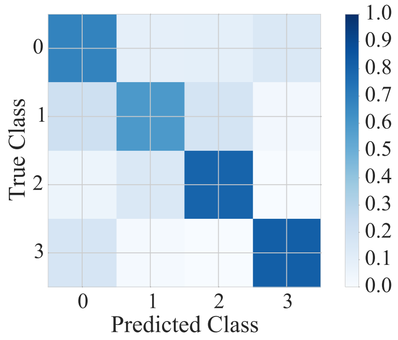

(c) $ASWS$ normalized confusion matrix

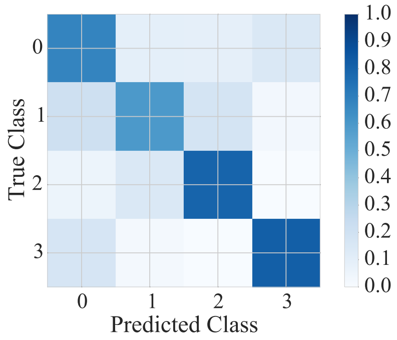

(d) $ASWS$ posterior probabilities

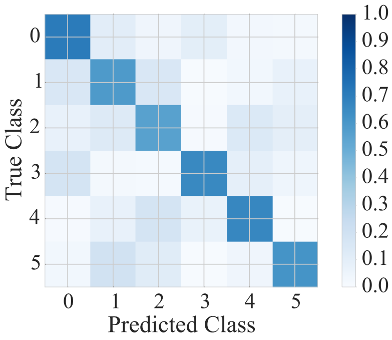

(e) *KSTS* normalized confusion matrix

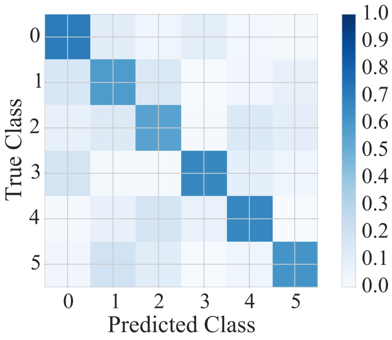

(f) *KSTS* posterior probabilities

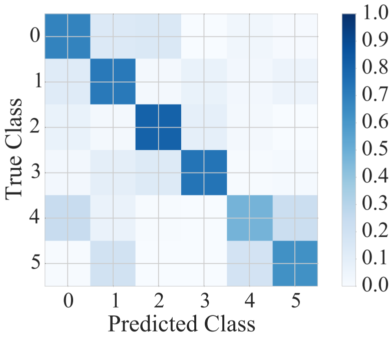

(g) *KSWS* normalized confusion matrix

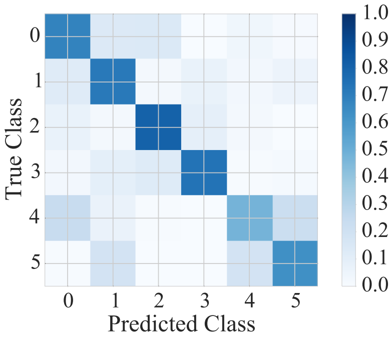

(h) *KSWS* posterior probabilities

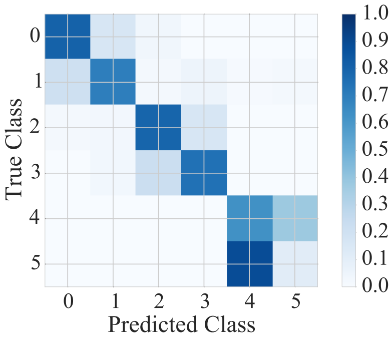

(i) *PS* normalized confusion matrix

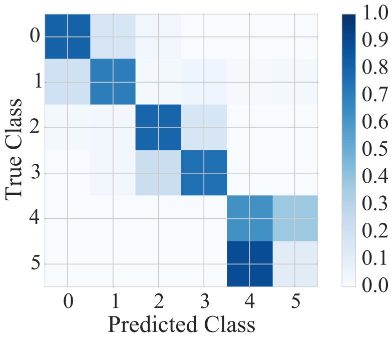

(j) *PS* posterior probabilities

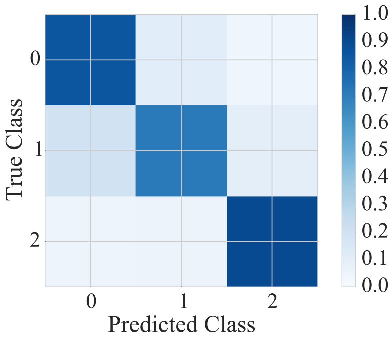

(k) *HS* normalized confusion matrix

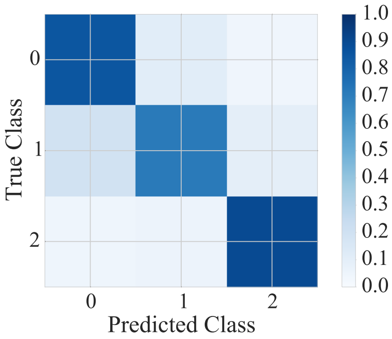

(l) *HS* posterior probabilities

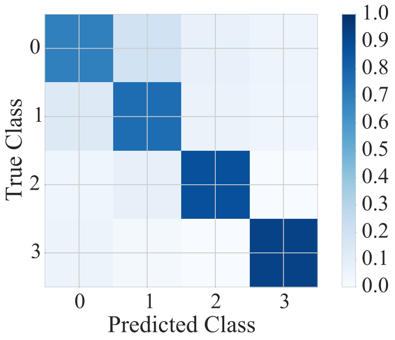

(m) *PC* normalized confusion matrix

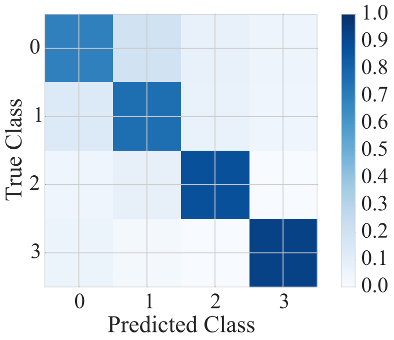

(n) *PC* posterior probabilities

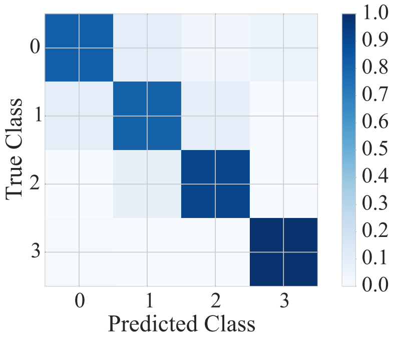

(o) *HC* normalized confusion matrix

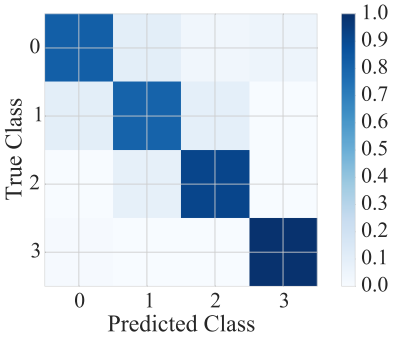

(p) *HC* posterior probabilities

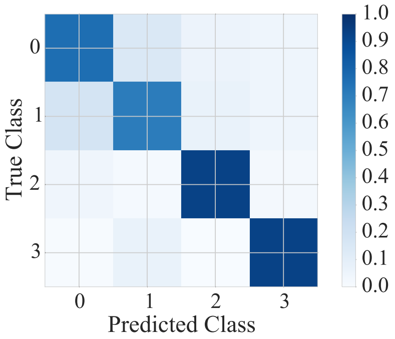

(q) *PT* normalized confusion matrix

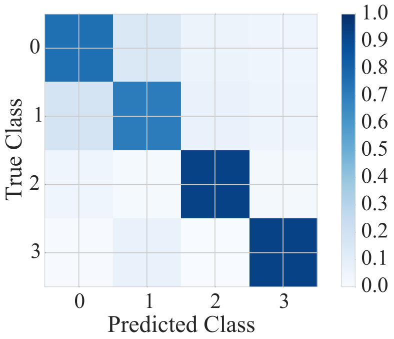

(r) *PT* posterior probabilities

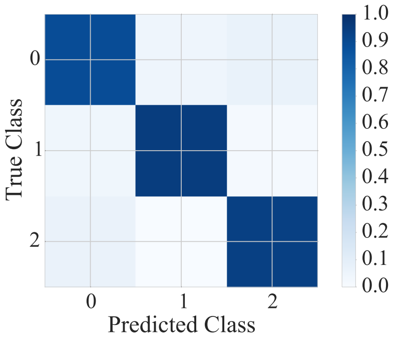

(s) *HT* normalized confusion matrix

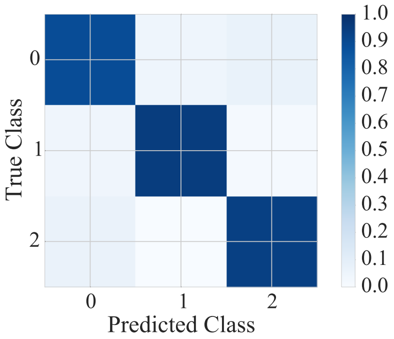

(t) *HT* posterior probabilities

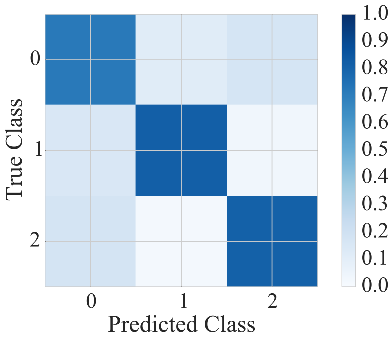

(u) *FT* normalized confusion matrix

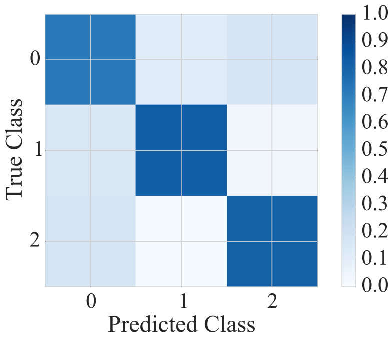

(v) *FT* posterior probabilities

Figure 1: Normalized confusion matrix (left column) and posterior probabilities (right column) for all features as input for different classification tasks, obtained by applying NB classifier (hypothesis 2a).

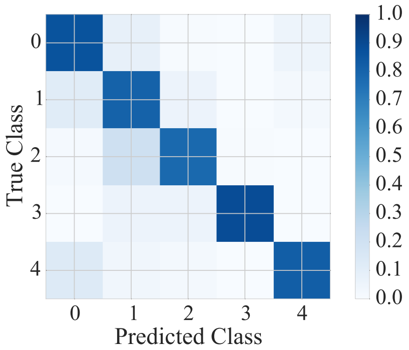

(a) $ASTS$ normalized confusion matrix

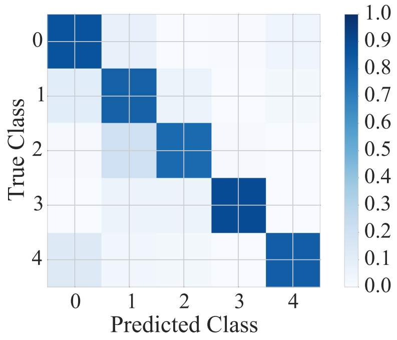

(b) $ASTS$ posterior probabilities

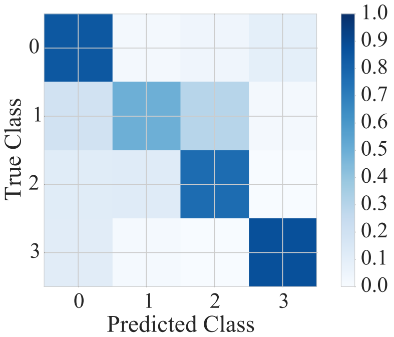

(c) $ASWS$ normalized confusion matrix

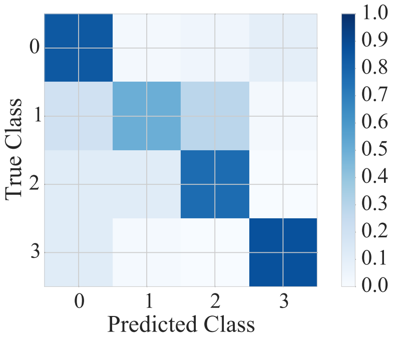

(d) $ASWS$ posterior probabilities

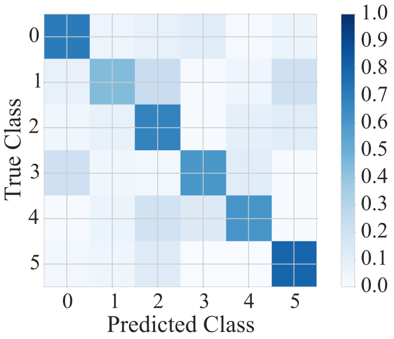

(e) $KSTS$ normalized confusion matrix

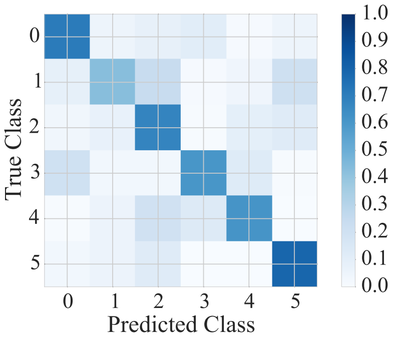

(f) $KSTS$ posterior probabilities

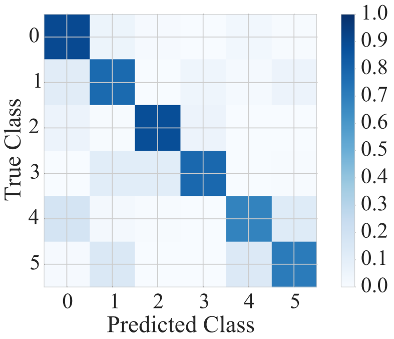

(g) $KSWS$ normalized confusion matrix

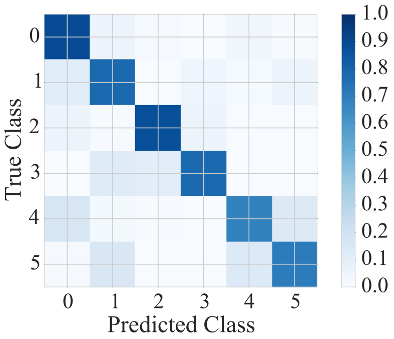

(h) $KSWS$ posterior probabilities

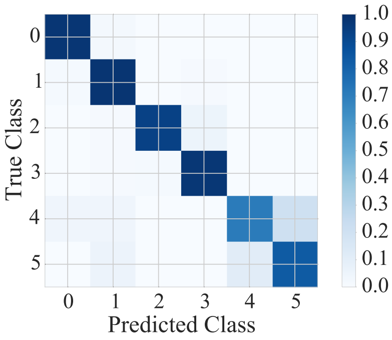

(i) PS normalized confusion matrix

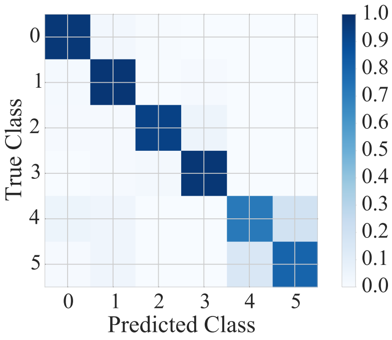

(j) PS posterior probabilities

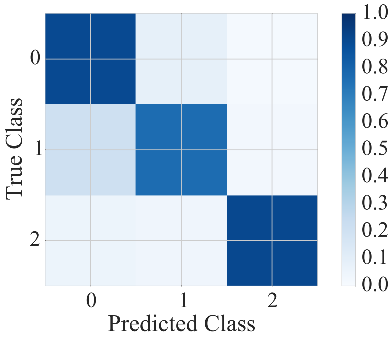

(k) HS normalized confusion matrix

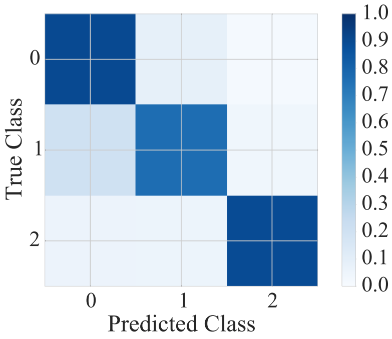

(l) HS posterior probabilities

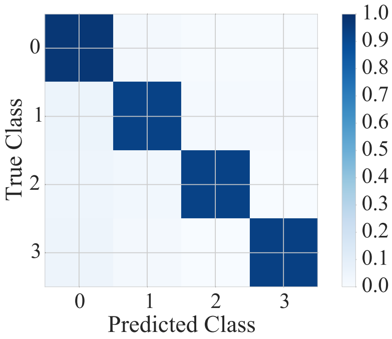

(m) PC normalized confusion matrix

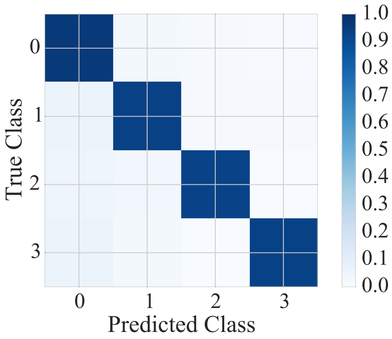

(n) PC posterior probabilities

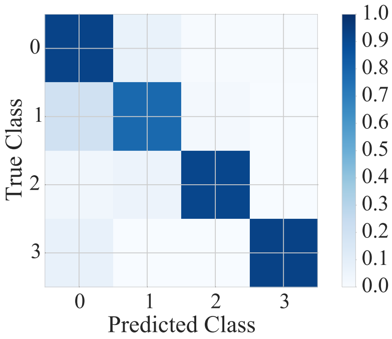

(o) HC normalized confusion matrix

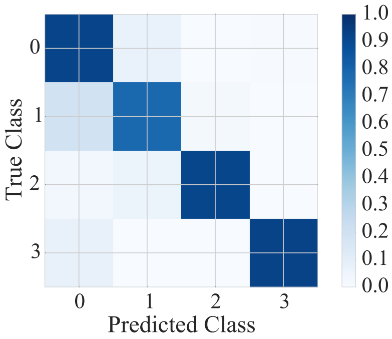

(p) HC posterior probabilities

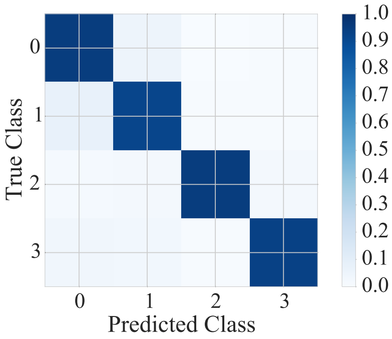

(q) *PT* normalized confusion matrix

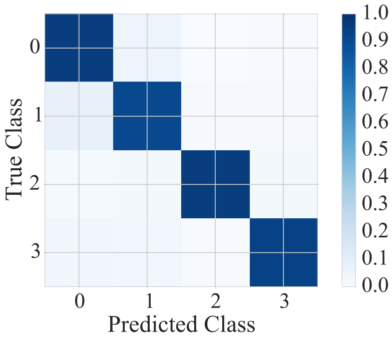

(r) *PT* posterior probabilities

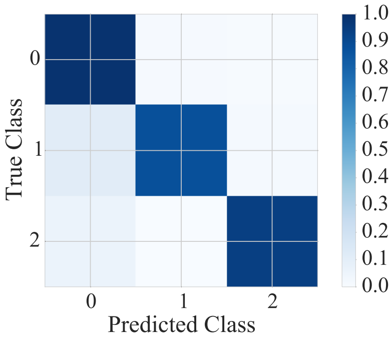

(s) *HT* normalized confusion matrix

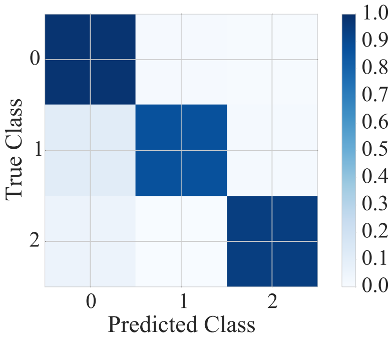

(t) *HT* posterior probabilities

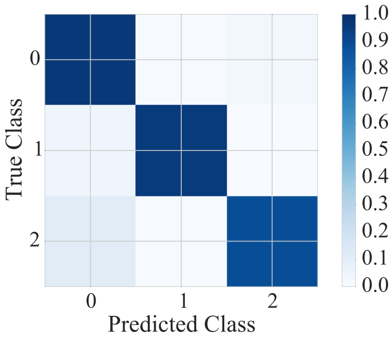

(u) *FT* normalized confusion matrix

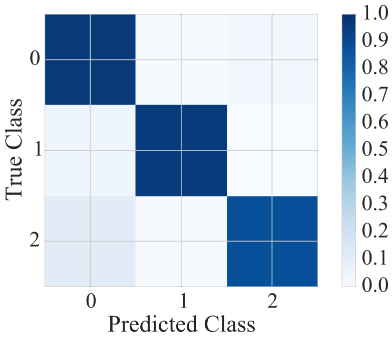

(v) *FT* posterior probabilities

Figure 2: Normalized confusion matrix (left column) and posterior probabilities (right column) for all features as input for different classification tasks, obtained by applying LR classifier (hypothesis 2a).

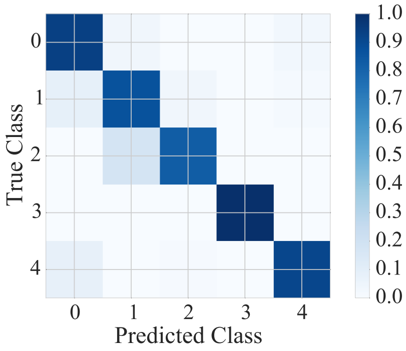

(a) $ASTS$ normalized confusion matrix

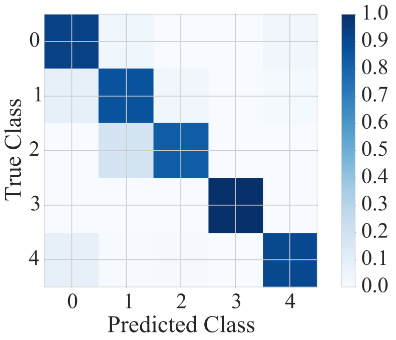

(b) $ASTS$ posterior probabilities

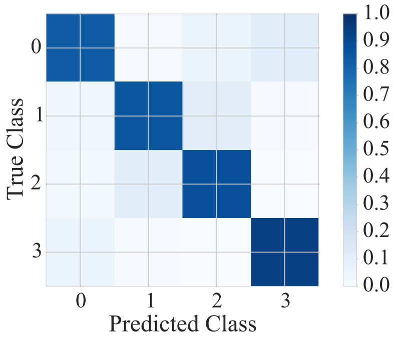

(c) $ASWS$ normalized confusion matrix

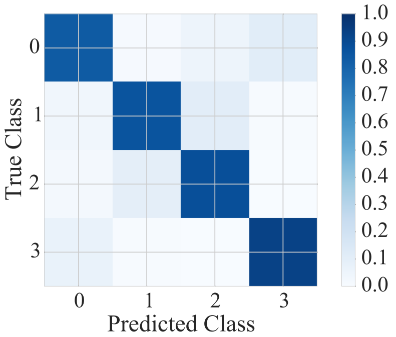

(d) $ASWS$ posterior probabilities

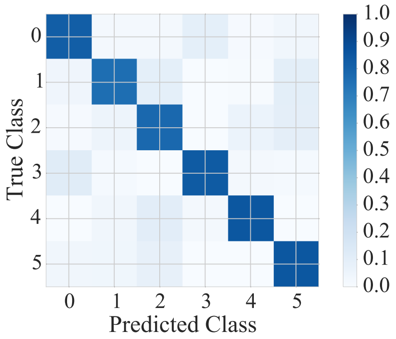

(e) $KSTS$ normalized confusion matrix

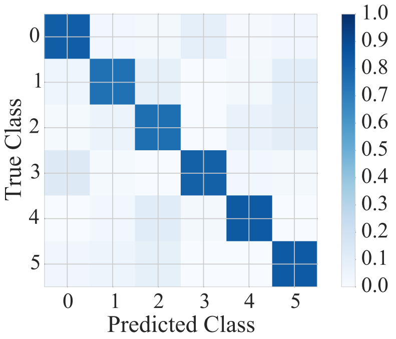

(f) $KSTS$ posterior probabilities

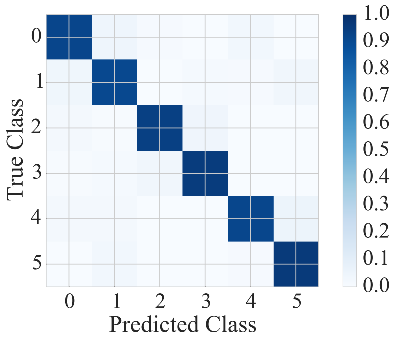

(g) $KSWS$ normalized confusion matrix

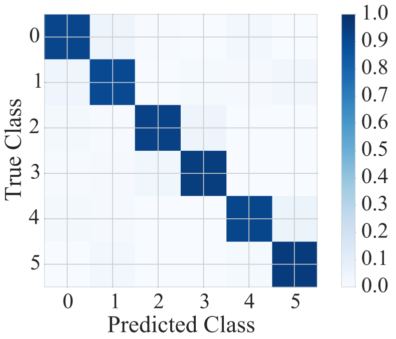

(h) $KSWS$ posterior probabilities

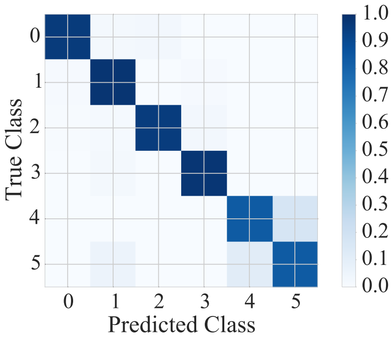

(i) *PS* normalized confusion matrix

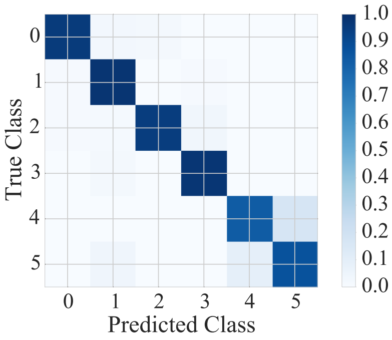

(j) *PS* posterior probabilities

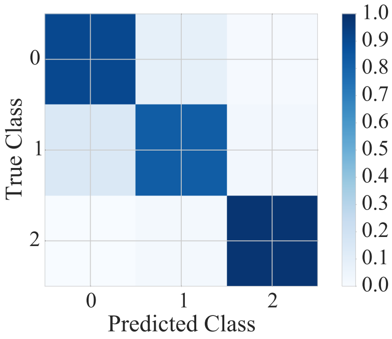

(k) *HS* normalized confusion matrix

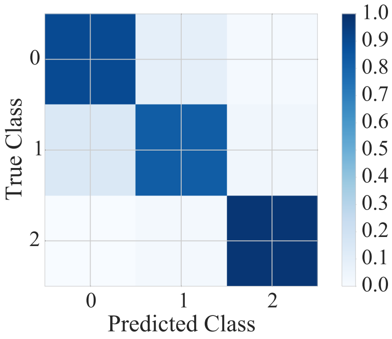

(l) *HS* posterior probabilities

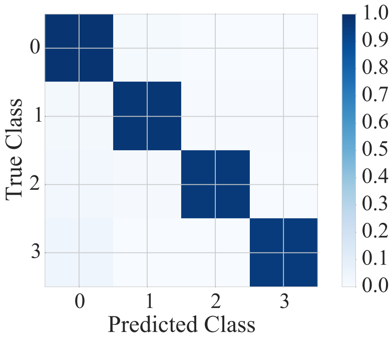

(m) *PC* normalized confusion matrix

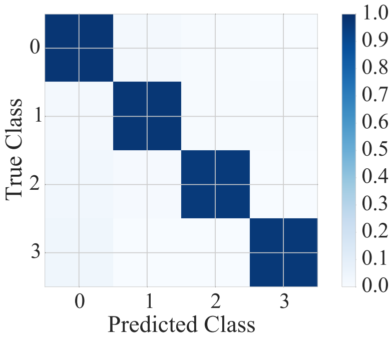

(n) *PC* posterior probabilities

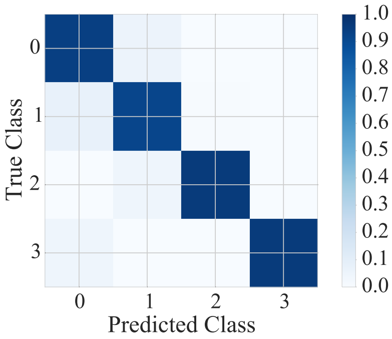

(o) *HC* normalized confusion matrix

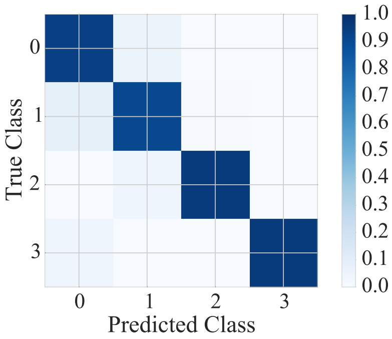

(p) *HC* posterior probabilities

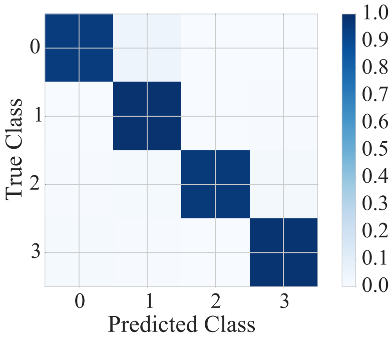

(q) *PT* normalized confusion matrix

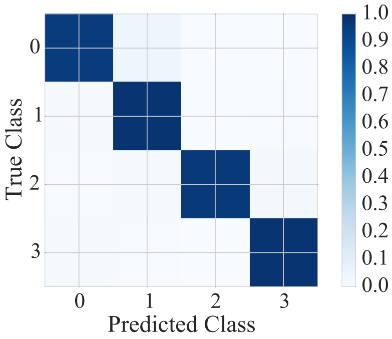

(r) *PT* posterior probabilities

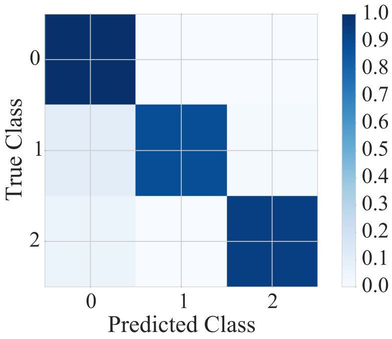

(s) *HT* normalized confusion matrix

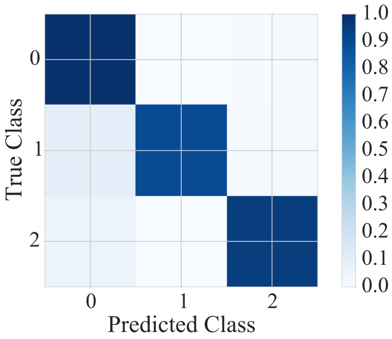

(t) *HT* posterior probabilities

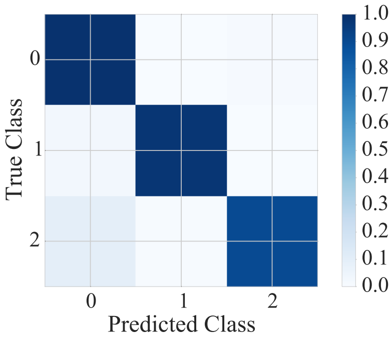

(u) *FT* normalized confusion matrix

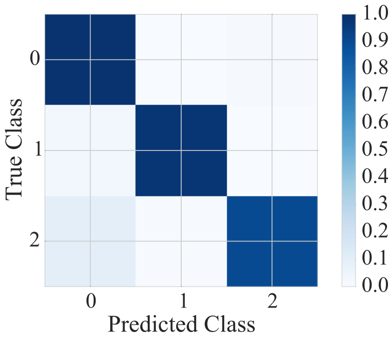

(v) *FT* posterior probabilities

Figure 3: Normalized confusion matrix (left column) and posterior probabilities (right column) for feature selection combined with the classification tasks, obtained by applying NB classifier (hypothesis 2a).

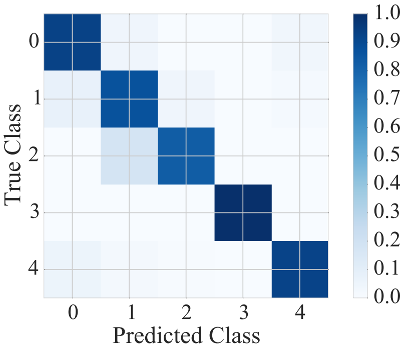

(a) $ASTS$ normalized confusion matrix

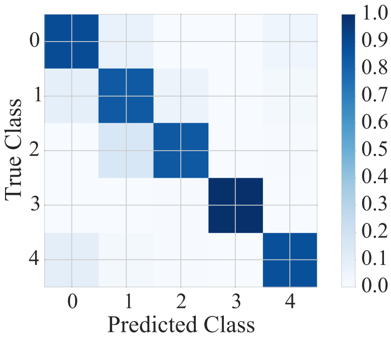

(b) $ASTS$ posterior probabilities

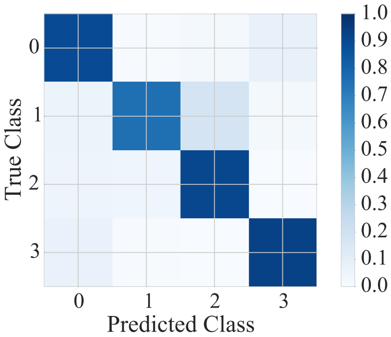

(c) $ASWS$ normalized confusion matrix

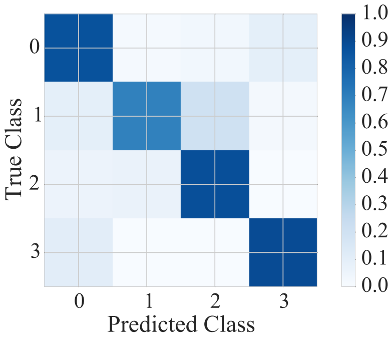

(d) $ASWS$ posterior probabilities

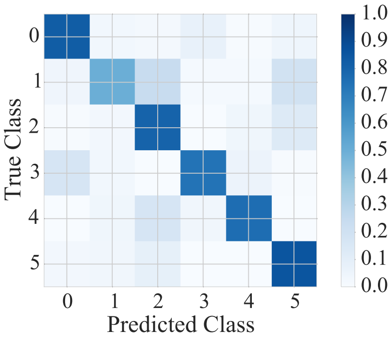

(e) $KSTS$ normalized confusion matrix

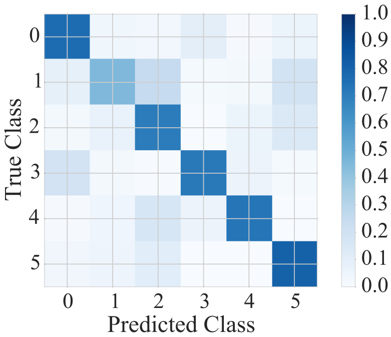

(f) $KSTS$ posterior probabilities

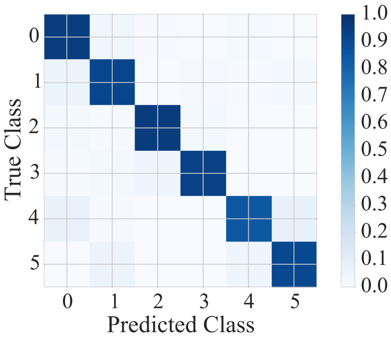

(g) $KSWS$ normalized confusion matrix

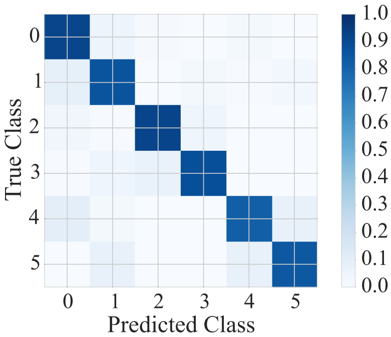

(h) $KSWS$ posterior probabilities

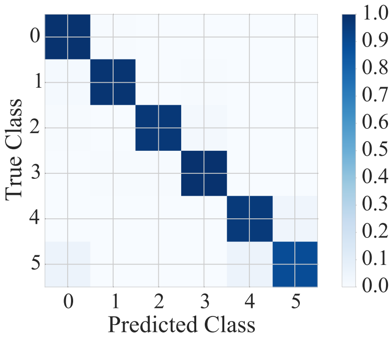

(i) PS normalized confusion matrix

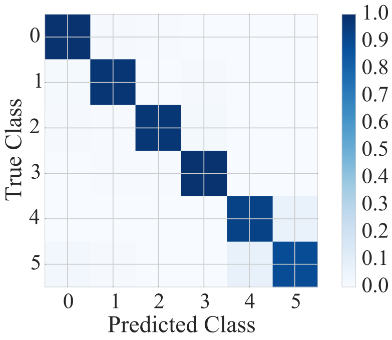

(j) PS posterior probabilities

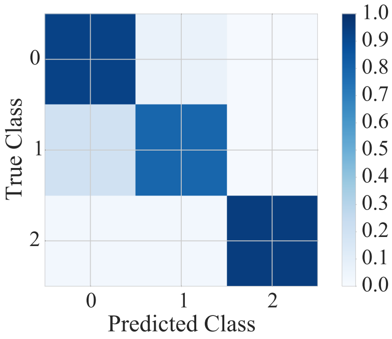

(k) HS normalized confusion matrix

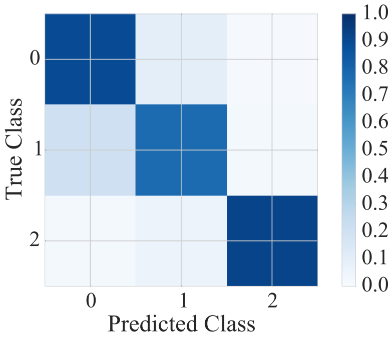

(l) HS posterior probabilities

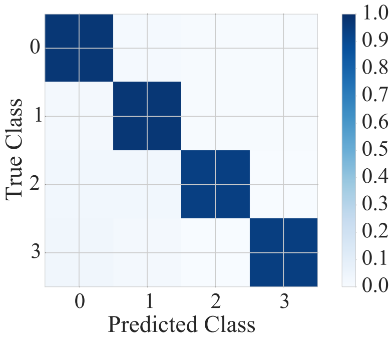

(m) PC normalized confusion matrix

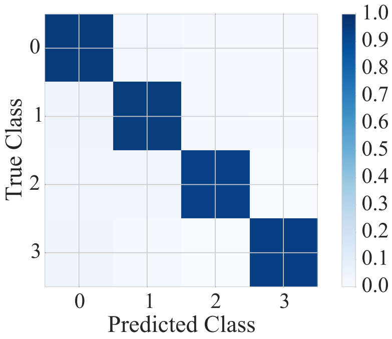

(n) PC posterior probabilities

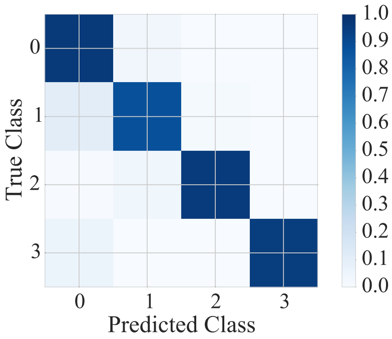

(o) HC normalized confusion matrix

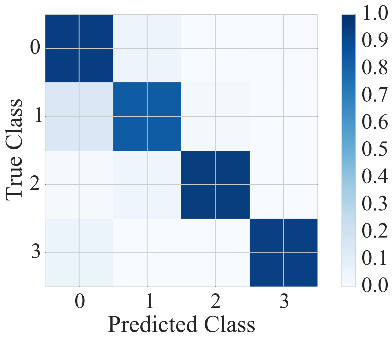

(p) HC posterior probabilities

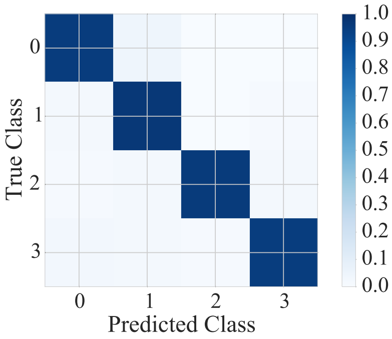

(q) *PT* normalized confusion matrix

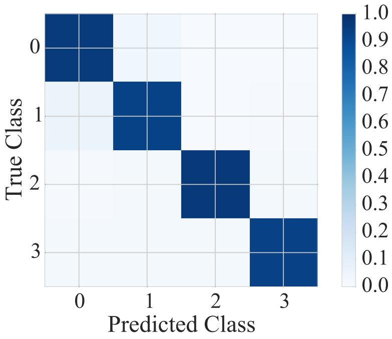

(r) *PT* posterior probabilities

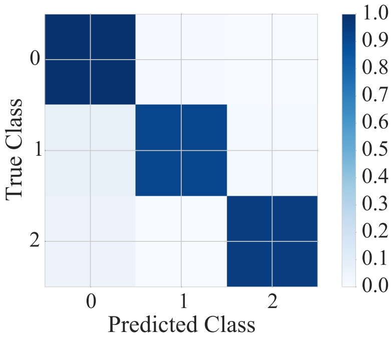

(s) *HT* normalized confusion matrix

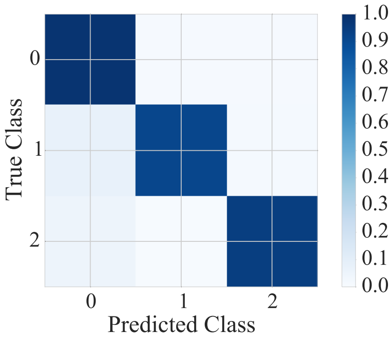

(t) *HT* posterior probabilities

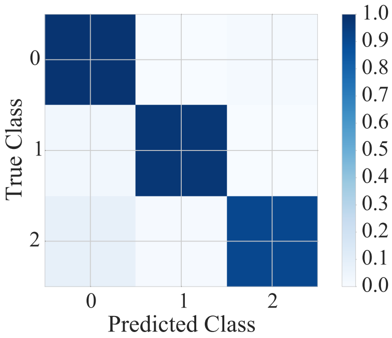

(u) *FT* normalized confusion matrix

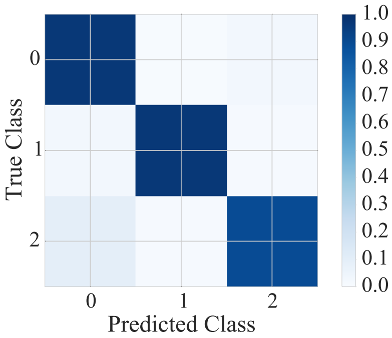

(v) *FT* posterior probabilities

Figure 4: Normalized confusion matrix (left column) and posterior probabilities (right column) for feature selection combined with the classification tasks, obtained by applying LR classifier (hypothesis 2a).
