# Supplementary material for: Does expert knowledge improve automatic probabilistic classification of gait joint motion patterns in children with cerebral palsy?
Source: PLoS One. 2017 Jun 1;12(6):e0178378. doi: 10.1371/journal.pone.0178378 (PMC5453476; doi:10.1371/journal.pone.0178378)
Supplement: S3 Appendix — (PDF) [file pone.0178378.s003.pdf]

Supplemental Material 2: Classification results for hypothesis 2b

This supplemental material provides the normalized confusion matrices and posterior probabilities for all classification tasks performed using Naive Bayes (NB3, Fig.1) and Logistic Regression (LR3, Fig.2) with the expert-defined but automatically discretized (NB3) or continuous (LR3) features (hypothesis 2b).

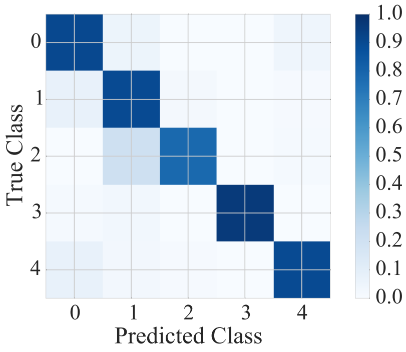

(a) *ASTS* normalized confusion matrix

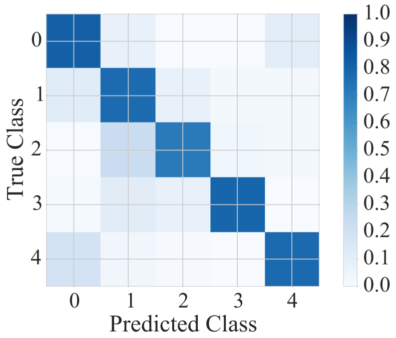

(b) *ASTS* posterior probabilities

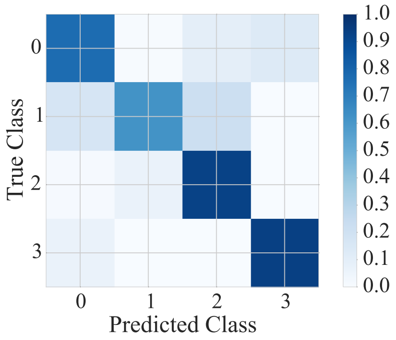

(c) *ASWS* normalized confusion matrix

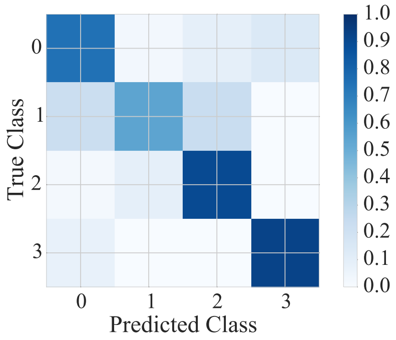

(d) *ASWS* posterior probabilities

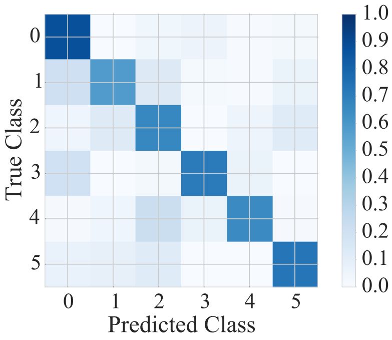

(e) $KSTS$ normalized confusion matrix

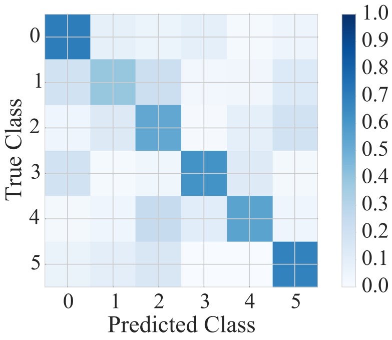

(f) $KSTS$ posterior probabilities

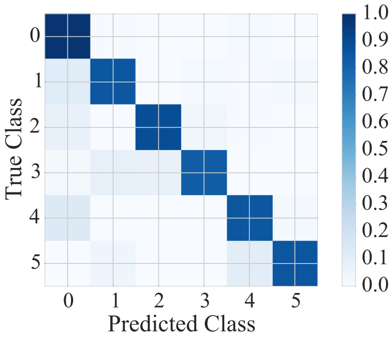

(g) $KSWS$ normalized confusion matrix

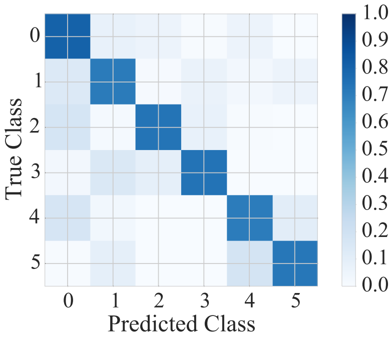

(h) $KSWS$ posterior probabilities

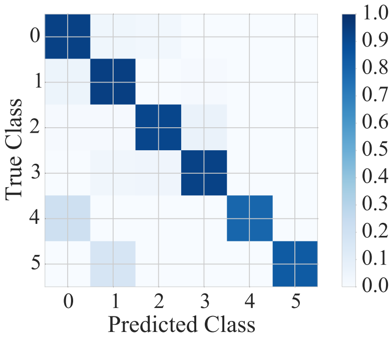

(i) PS normalized confusion matrix

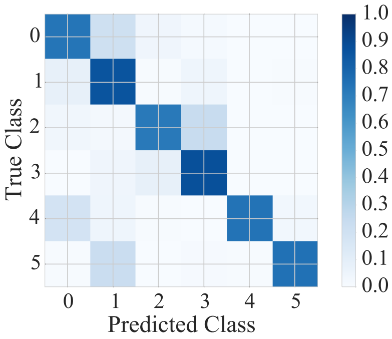

(j) PS posterior probabilities

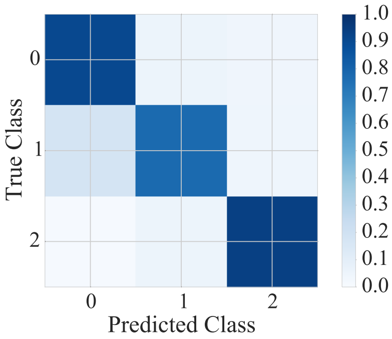

(k) HS normalized confusion matrix

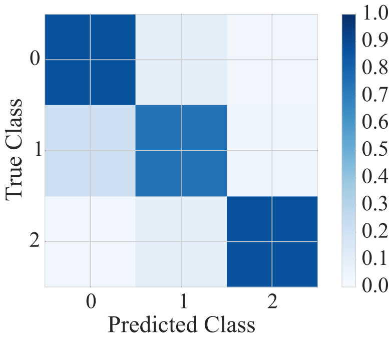

(l) HS posterior probabilities

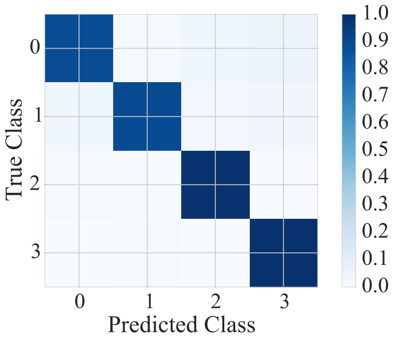

(m) *PC* normalized confusion matrix

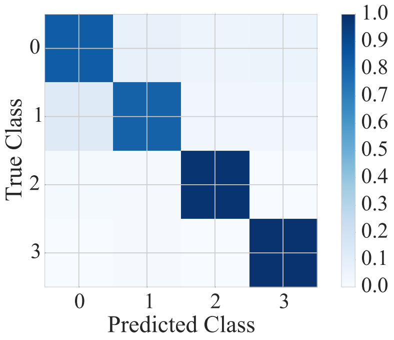

(n) *PC* posterior probabilities

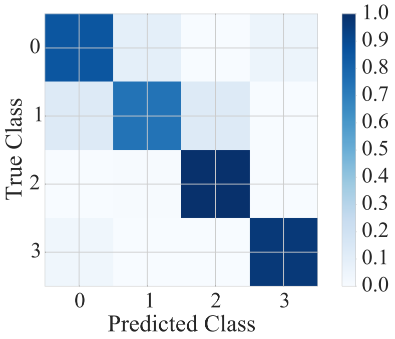

(o) *HC* normalized confusion matrix

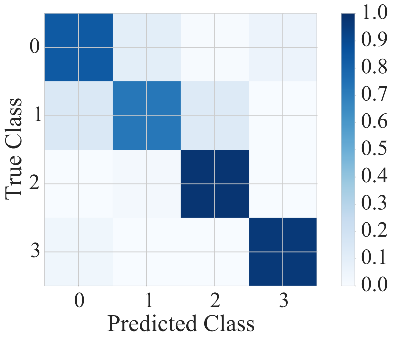

(p) *HC* posterior probabilities

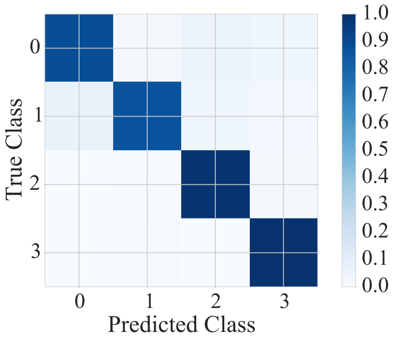

(q) *PT* normalized confusion matrix

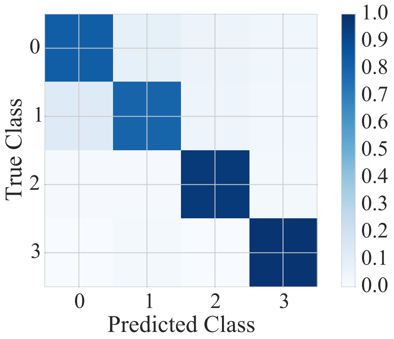

(r) *PT* posterior probabilities

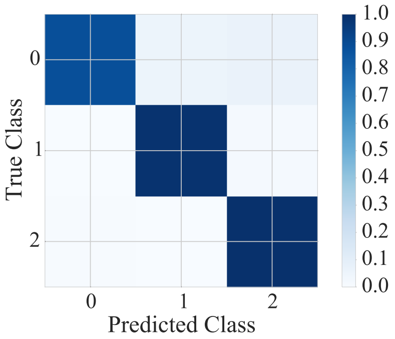

(s) *HT* normalized confusion matrix

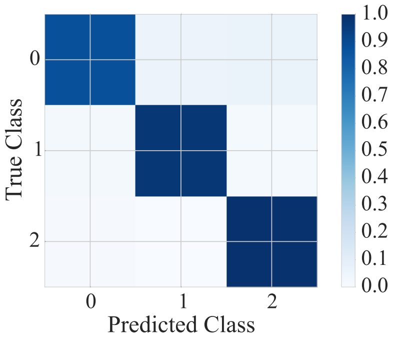

(t) *HT* posterior probabilities

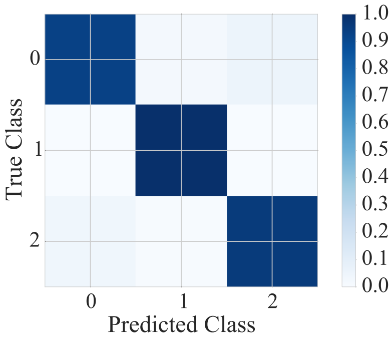

(u) FT normalized confusion matrix

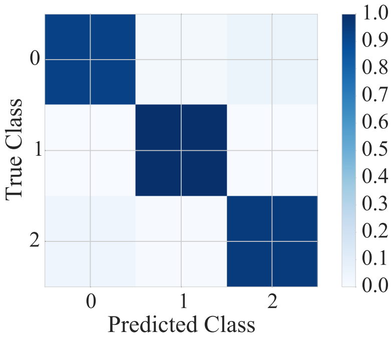

(v) FT posterior probabilities

Figure 1: Normalized confusion matrix (left column) and posterior probabilities (right column) for expert-defined but automatically discretized features as input for different classification tasks, obtained by applying Naive Bayes classifier (hypothesis 2b).

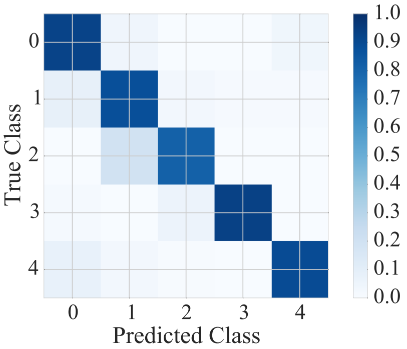

(a) $ASTS$ normalized confusion matrix

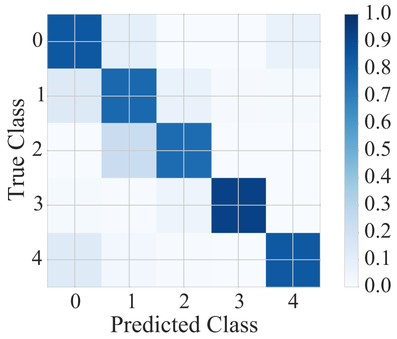

(b) $ASTS$ posterior probabilities

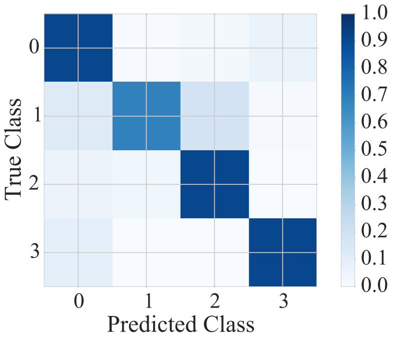

(c) $ASWS$ normalized confusion matrix

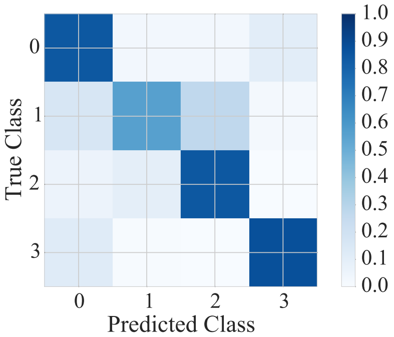

(d) $ASWS$ posterior probabilities

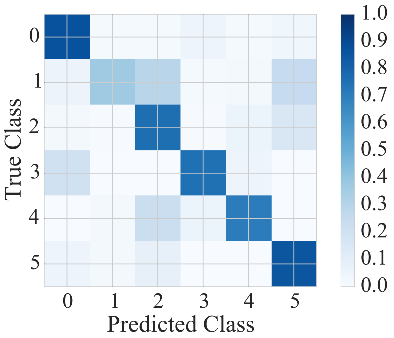

(e) $KSTS$ normalized confusion matrix

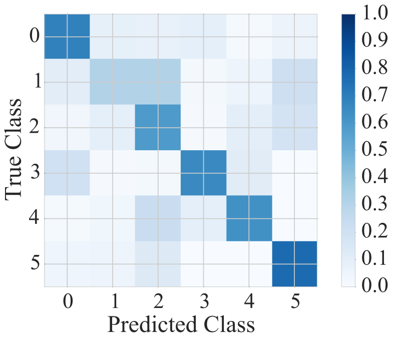

(f) $KSTS$ posterior probabilities

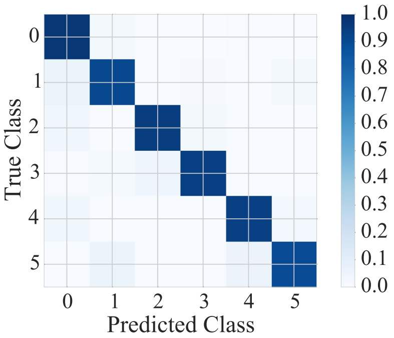

(g) $KSWS$ normalized confusion matrix

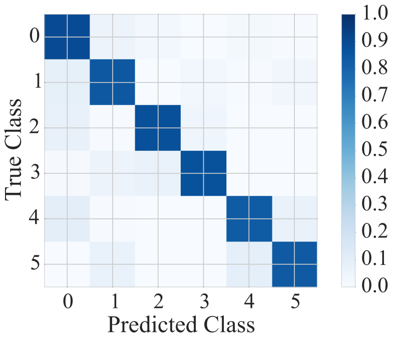

(h) $KSWS$ posterior probabilities

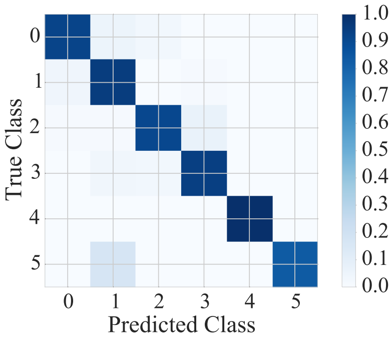

(i) PS normalized confusion matrix

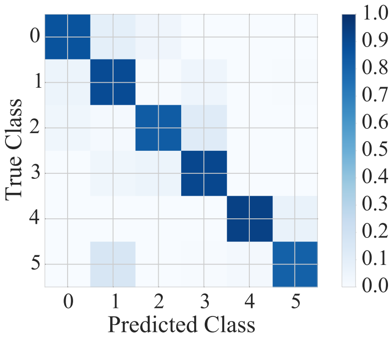

(j) PS posterior probabilities

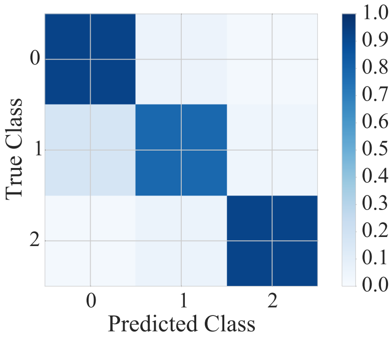

(k) HS normalized confusion matrix

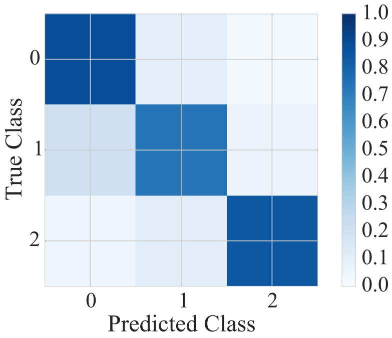

(l) HS posterior probabilities

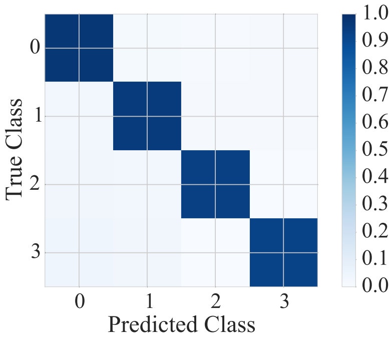

(m) PC normalized confusion matrix

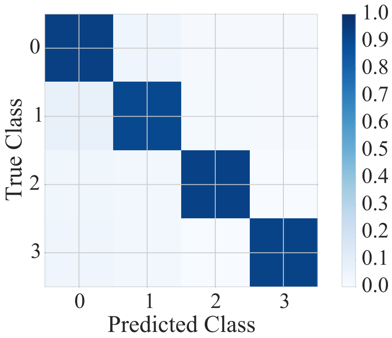

(n) PC posterior probabilities

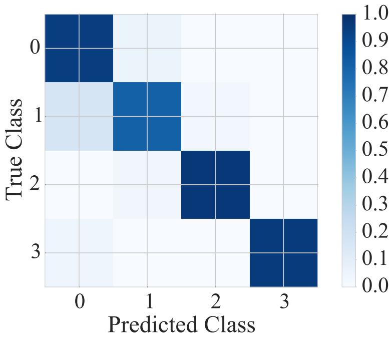

(o) HC normalized confusion matrix

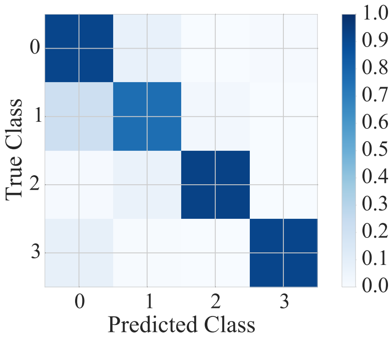

(p) HC posterior probabilities

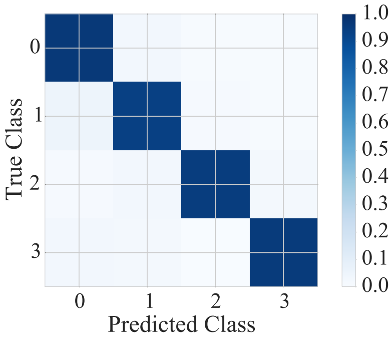

(q) *PT* normalized confusion matrix

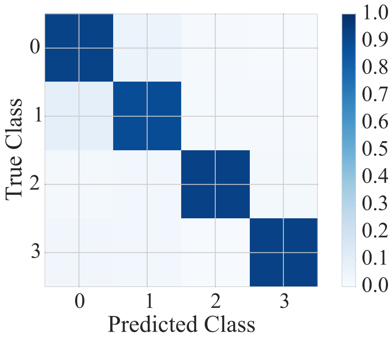

(r) *PT* posterior probabilities

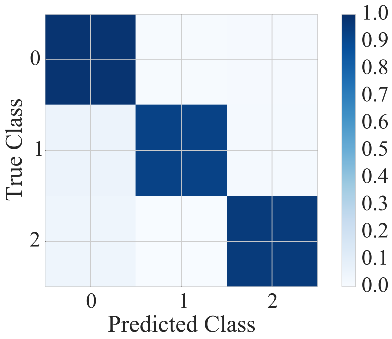

(s) *HT* normalized confusion matrix

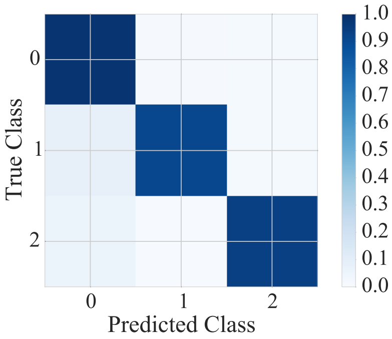

(t) *HT* posterior probabilities

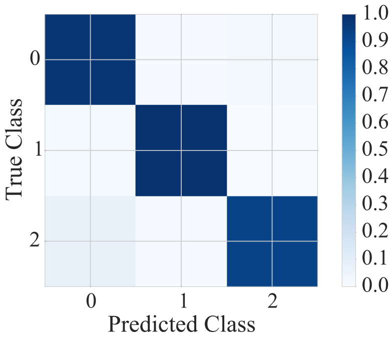

(u) *FT* normalized confusion matrix

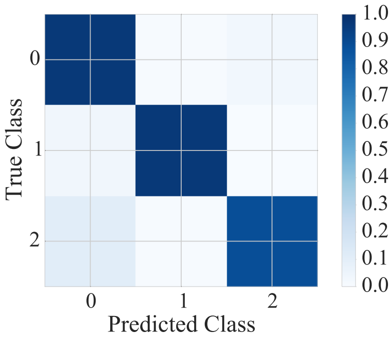

(v) *FT* posterior probabilities

Figure 2: Normalized confusion matrix (left column) and posterior probabilities (right column) for expert-defined continuous features as input for different classification tasks, obtained by applying Logistic Regression classifier (hypothesis 2b).
